# Gut symbiotic bacteria are involved in nitrogen recycling in the tephritid fruit fly *Bactrocera dorsalis*

**DOI:** 10.1186/s12915-022-01399-9

**Published:** 2022-09-14

**Authors:** Xueming Ren, Shuai Cao, Mazarin Akami, Abdelaziz Mansour, Yishi Yang, Nan Jiang, Haoran Wang, Guijian Zhang, Xuewei Qi, Penghui Xu, Tong Guo, Changying Niu

**Affiliations:** Hubei Key Laboratory of Insect Resource Application and Sustainable Pest Control, College of Plant Science & Technology, Huazhong Agricultural University, Wuhan, 430070 China

**Keywords:** *Bactrocera dorsalis*, Nitrogenous waste recycling, Biological nitrogen fixation, Amino acid biosynthesis, Urease activity

## Abstract

**Background:**

Nitrogen is considered the most limiting nutrient element for herbivorous insects. To alleviate nitrogen limitation, insects have evolved various symbiotically mediated strategies that enable them to colonize nitrogen-poor habitats or exploit nitrogen-poor diets. In frugivorous tephritid larvae developing in fruit pulp under nitrogen stress, it remains largely unknown how nitrogen is obtained and larval development is completed.

**Results:**

In this study, we used metagenomics and metatranscriptomics sequencing technologies as well as in vitro verification tests to uncover the mechanism underlying the nitrogen exploitation in the larvae of *Bactrocera dorsalis*. Our results showed that nitrogenous waste recycling (NWR) could be successfully driven by symbiotic bacteria, including Enterobacterales, Lactobacillales, Orbales, Pseudomonadales, Flavobacteriales, and Bacteroidales. In this process, urea hydrolysis in the larval gut was mainly mediated by *Morganella morganii* and *Klebsiella oxytoca*. In addition, core bacteria mediated essential amino acid (arginine excluded) biosynthesis by ammonium assimilation and transamination.

**Conclusions:**

Symbiotic bacteria contribute to nitrogen transformation in the larvae of *B. dorsalis* in fruit pulp. Our findings suggest that the pattern of NWR is more likely to be applied by *B. dorsalis*, and *M. morganii*, *K. oxytoca*, and other urease-positive strains play vital roles in hydrolysing nitrogenous waste and providing metabolizable nitrogen for *B. dorsalis*.

**Supplementary Information:**

The online version contains supplementary material available at 10.1186/s12915-022-01399-9.

## Background

Nitrogen is generally considered the most limiting nutrient element for herbivorous insects [1–3]. As nitrogen is a major structural component of insect tissues, insects require and contain far more nitrogen than plants. However, nitrogen utilization in insects is lower because of the excretion of large quantities of nitrogenous waste products [1, 4]. When dietary nitrogen becomes scarce in the environment, opportunistic feeders must seek out large amounts of it to meet their requirements for growth and reproduction.

To alleviate nitrogen limitation, insects have evolved various symbiotically mediated strategies that enable them to colonize nitrogen-poor habitats or exploit nitrogen-poor diets [5]. Some herbivores acquire additional nitrogen from symbiotic bacteria through biological nitrogen fixation (BNF); that is, atmospheric nitrogen is fixed via diazotrophic bacteria to provide the host with available nitrogen. Most BNF reactions occur in different gut regions of insect hosts such as termites, tephritid fruit flies, xylophagous beetles, weevils, and click beetles [6–9]. In addition to occurring within the body, BNF also plays integral roles in the fungal gardens of leaf-cutter ants to supplement the nitrogen budget of ants [10, 11]. However, some insects employ nitrogenous waste recycling (NWR) as a key way of acquiring supplemental nitrogen sources, in which, nitrogenous wastes are employed as a metabolic substrate of core symbionts (including bacteria, fungi and other microbiota members) to synthesize essential amino acids (EAAs) that can be reabsorbed by insect hosts [12]. NWR occurs in cockroach-*Blattabacterium* symbioses, shield bug-*Erwinia*-like bacterium partnerships, bean bug-*Burkholderia* associations, carpenter ant-*Blochmannia* partnerships, and turtle ant-core bacterium associations [13–17]. Recent studies have shown that many obligate symbionts associated with NWR, especially in hemipterans, possess greatly reduced genomes due to independent gene losses across lineages [18–21]. In these cases, NWR is not mediated by independent obligate symbionts but by host-symbiont complexes. For instance, aphid transaminases incorporate ammonia-derived nitrogen into carbon skeletons synthesized by *Buchnera* to generate EAAs [12]. In a similar context, other plant-sucking insects, such as brown planthoppers, cicadas, and mealybugs, also build associations with obligate symbionts to compensate for deficiencies in essential amino acids or other nutrients in plant sap [22–24]. BNF and NWR reactions can also function together within a species, as observed in certain termites [25], cockroaches [26], bark beetles [27], longhorned beetles [28], and cochineal carmine [29, 30], to compensate for extremely low dietary nitrogen levels.

Tephritid fruit flies are economically important pests that threaten horticultural and fruit production around the world [31, 32]. Adult flies are opportunistic feeders with the ability to fly and forage for plant-derived exudates, extrafloral nectaries, pollen, fruit juice, ripe fruits, microorganisms, honeydew, and bird droppings (considered their primary nitrogen source) [31, 33]. Their larvae are frugivorous and live in relatively confined spaces [34]. During larval development, the larvae feed on large amounts of fruit pulp to achieve an optimal increase in biomass within a few days [35]. Nitrogen exploitation in tephritid fruit fly pests has been studied in adult Queensland fruit flies, Mediterranean fruit flies, and olive fruit flies [7, 33, 36]. The results showed that Enterobacteriaceae in the guts of adults are hidden players in nitrogen acquisition by BNF in Queensland fruit flies and Mediterranean fruit flies and enable olive flies to exploit urea as an available nitrogen source. However, the extents of low nitrogen stress faced by adults and larvae are very different. Most fruits have a high sugar content but low nitrogen content [31, 37, 38]. Moreover, the limited mobility of larvae makes it difficult for them to find an alternative host [39, 40]. These factors remain a tremendous challenge in resource exploitation by frugivorous larvae in nitrogen-poor environments. The rapid deterioration of fruits caused by fruit fly invasion is always associated with the proliferation of abundant microbiota, which may contribute to the flies’ nitrogen and carbon metabolism [41]. We therefore hypothesized that symbiotic bacteria contribute to nitrogen transformation in frugivorous larvae in this enclosed niche. To test our hypothesis, we explored the underlying mechanism of nitrogen exploitation in *Bactrocera dorsalis*, a notorious tephritid insect pest regarded as one of the most damaging fruit flies, infesting more than 250 different fruits and vegetables [32].

In our study, metagenomics (DNA-seq) and metatranscriptomics analyses (RNA-seq) were used to evaluate the composition and diversity of bacterial communities across life stages, and the potential functions of core bacteria (Several symbiotic bacteria with relatively high abundance and expression) involved in nitrogen exploitation were also investigated. Due to their abundance of the Enterobacteriaceae species *Morganella morganii* and *Klebsiella oxytoca* and their involvement in nitrogen metabolic pathways, as shown in this study, they are regarded as major actors in nitrogen recycling, nutritional provisioning, and urea metabolism in *B. dorsalis* larvae. The results of this work confirm their ecological relevance, which could be exploited in mass rearing for the application of the sterile insect technique (SIT) to control target pests and reduce their devastating occurrence in crops and vegetables.

## Results

### The composition and diversity of bacterial communities in *B. dorsalis*

**Fig. 1:**
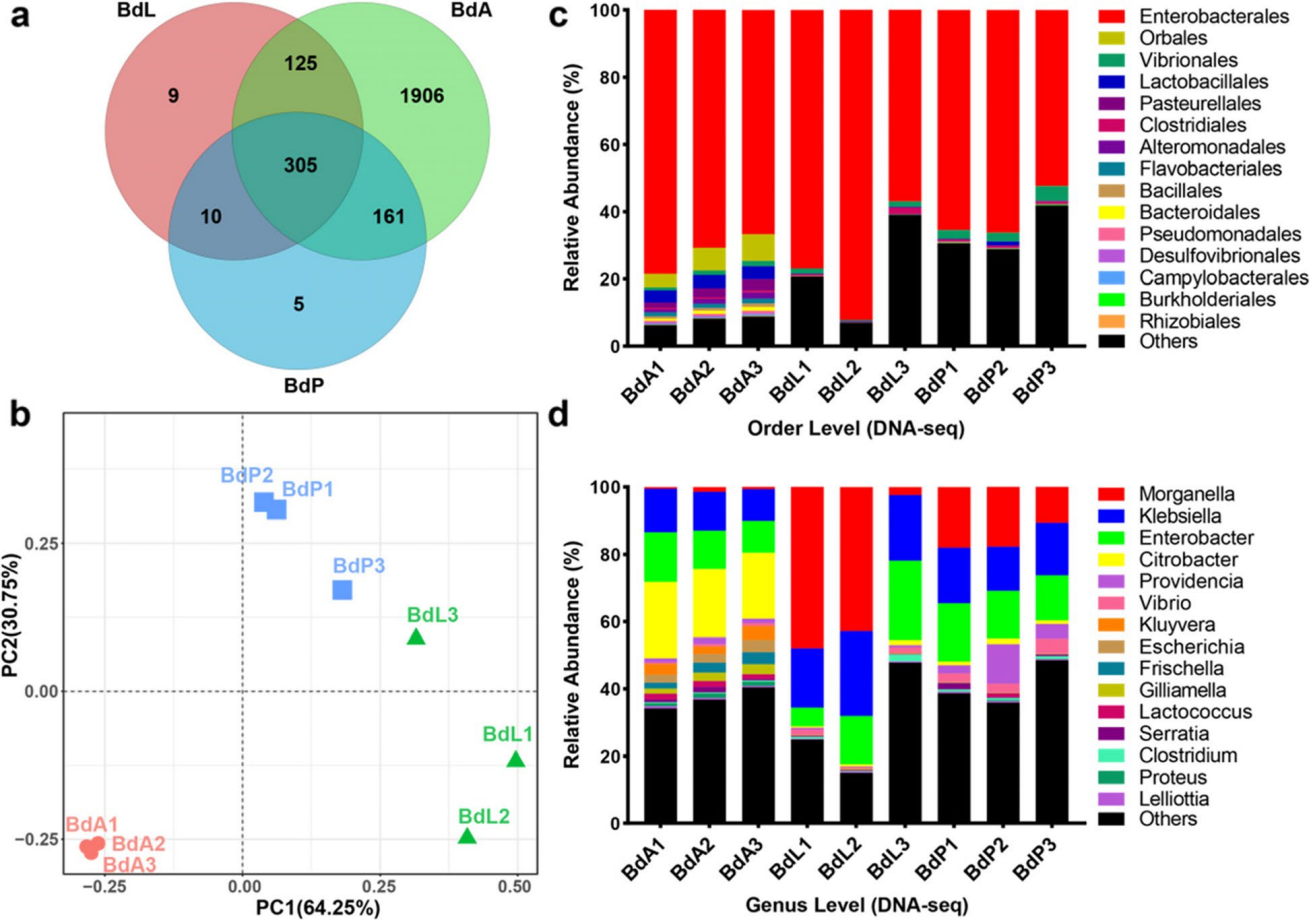
Metagenomic data analysis (only bacteria). **a** Venn diagram of the numbers of annotated bacteria in different life stages. **b** PCoA of the composition of bacteria across different life stages. **c** The composition of bacteria at the order level. **d** The composition of bacteria at the genus level. BdA: adults; BdL: larvae; BdP: pupae

A total of 1,516,550 scaffolds were constructed from nine metagenomic libraries (Additional file 1: Table S1). Among the annotated species, bacteria were identified as the most abundant community (approximately 82.30%). A Venn diagram showed that the number of annotated bacteria in adult flies (2,497 species) was far greater than those in larvae (449 species) and pupae (481 species), and 305 bacteria were found in *B. dorsalis* across all life stages (Fig. 1a). Principal coordinate analysis (PCoA, as implemented in QIIME) and alpha diversity analysis suggested that the bacterial communities in larvae and pupae had similar diversity, which was lower than that in adults (Fig. 1b, Additional file 1: Table S2).

Across different life stages, Proteobacteria was the most abundant phylum (adult 88.40 ± 0.68%, larva 77.53 ± 10.08%, pupa 65.90 ± 4.01%), followed by Firmicutes and Bacteroidetes. The order Enterobacterales within the phylum Proteobacteria was dominant in each life stage, representing 61.34 ± 4.51% to 75.33 ± 10.21% of the total bacterial population. Beyond Enterobacterales, the most common bacteria from Orbales, Lactobacillales, and Pasteurellales were mainly present in adults, while the orders Vibrionales and Clostridiales were mainly detected in larvae and pupae. At the genus level, *Citrobacter* (20.93 ± 0.95% in adults) and *Morganella* (31.05 ± 4.40% in larvae, 15.40 ± 2.42% in pupae) represented the most abundant genera across different life stages. Additionally, *Klebsiella* (11.26 ± 1.03% in adults, 20.81 ± 2.28% in larvae, 15.15 ± 1.01% in pupae) and *Enterobacter* (11.87 ± 1.54% in adults, 14.48 ± 5.21% in larvae, 14.96 ± 1.18% in pupae) were found in high proportions across experimental samples (Fig. 1c, d, Additional file 1: Table S3).

### Expression levels of functional bacteria and pathways in *B. dorsalis*

**Fig. 2:**
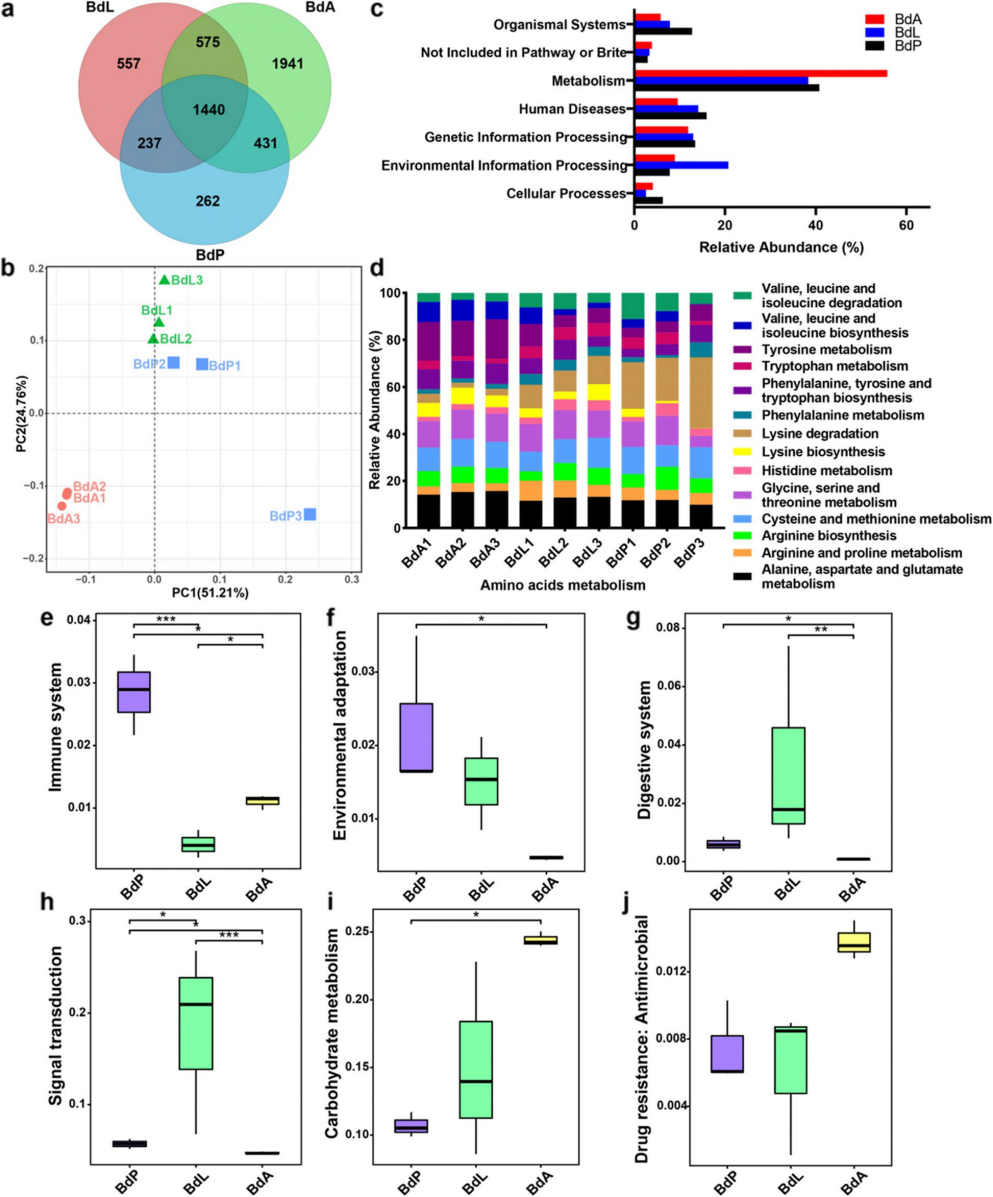
Metatranscriptomic data analysis. **a** Venn diagram of the number of annotated KOs in different life stages. **b** PCoA of the composition of KOs across different life stages. **c** Annotation results of first-order metabolic pathways based on KEGG. **d** The composition of pathways involved in amino acid metabolism. **e–j** Annotation results of second-order metabolic pathways based on KEGG (**e** immune system, **f** environmental adaptation, **g** digestive system, **h** signal transduction, **i** carbohydrate metabolism, **j** drug resistance). Error bars represent the standard error of the mean. “*,” “**,” and “***” represent statistical significance at *p* < 0.05, 0.01, and 0.001, respectively. BdA: adults; BdL: larvae; BdP: pupae

The percentages of rRNA sequences in the clean datasets were 0.77 ± 0.03% in adults, 3.79 ± 0.61% in larvae, and 1.42 ± 0.07% in pupae. After removing rRNA sequences, a total of 602,416 full-length transcripts were generated from nine metatranscriptomics libraries (Additional file 1: Table S4), and 5443 unigene sets were annotated based on the Kyoto Encyclopedia of Genes and Genomes (KEGG). Consistent with the DNA-seq results mentioned above, the greatest number of KEGG orthology groups (KOs) was annotated in adults (4387 KOs, Fig. 2a). The compositions of KOs in larvae and pupae were similar but quite different from those in adults (Fig. 2b).

KOs belonging to the category of metabolism (first-order metabolic pathway) were the most abundant throughout the life stages (representing 40.80 ± 2.33, 38.36 ± 5.42, and 55.79 ± 0.52% of the total KO functions in gut bacteria of pupae, larvae and adults, respectively; Fig. 2c). A particular focus was placed on amino acid metabolism, belonging to the category of metabolism, in our study. The results showed that (1) the gut bacteria found in each life stage were involved in all the pathways of amino acid metabolism to varying degrees, and (2) KOs unique to alanine, aspartate, and glutamate metabolism (15.03 ± 0.46% in adults, 12.56 ± 0.53% in larvae, 11.13 ± 0.64% in pupae) were highly abundant throughout the life stages (Fig. 2d). In addition to amino acid metabolism, we found that KOs unique to pupal symbiotic bacteria were enriched in the immune system and environmental adaptation, whereas KOs of the digestive system and signal transduction were highly expressed in larvae, and KOs of carbohydrate or energy metabolism and drug resistance (antimicrobial) were abundant in adults (Fig. 2e–j, Additional file 1: Fig. S1).

The expression levels of gut bacteria were preliminarily analysed based on the RNA-seq data to help us predict the relationships between functional bacteria and specific pathways. The order Orbales (23.39 ± 1.03%) was the most abundant bacterial order in adults, and bacteria from Enterobacterales and Lactobacillales were highly abundant throughout the life stages. At the genus level, the relative abundance of the core bacteria varied among developmental stages. For instance, *Gilliamella* was dominant in the adult stage (10.40 ± 0.48%), followed by *Orbus* (5.32 ± 0.14%), *Lactococcus* (3.47 ± 0.38%), *Vagococcus* (3.29 ± 0.17%), and *Frischella* (3.06 ± 0.21%), whereas the most common genera in larvae were *Morganella* (2.67 ± 1.32%) and *Klebsiella* (1.11 ± 0.89%). *Providencia* (2.82 ± 0.44%), *Morganella* (1.09 ± 0.45%), and *Vibrio* (0.40 ± 0.07%) were dominant in pupae (Additional file 1: Table S5).

### Biological nitrogen fixation (BNF) is not a major contributor to the host nitrogen budget

The mean nitrogen:carbon (N:C) ratio in the *B. dorsalis* biomass was markedly higher than that in their natural diets (Additional file 1: Fig. S2), suggesting that nitrogen nutrient enrichment occurred in larvae and adults. To test whether BNF occurred in *B. dorsalis*, flies at different life stages were subjected to an acetylene reduction assay (ARA) according to the method previously used in Mediterranean fruit flies [7]. In our test, no ethylene could be detected in adults, larvae or pupae after incubation in acetylene for 0, 1, 2, 4, 8, and 16 h (Table 1). However, *K. oxytoca*, one of the most common bacteria at all developmental stages, produced 1.84 μl of ethylene per hour on average. Our results suggested that the existence of potential diazotrophic bacteria is not the determining factor in BNF induction in *B. dorsalis*. Furthermore, the contribution of BNF to nitrogen assimilation may not be very important in *B. dorsalis*.

**Table 1:** Acetylene reduction activity detected among in vivo and in vitro bacterial communities of *B. dorsalis*. Nitrogenase can reduce acetylene (C_2_H_2_) to ethylene (C_2_H_4_). No ethylene was detected in the three life stages investigated in this study

Reaction time (h)	Adults (*n*=6, μl/h)	Larvae (*n*=10, μl/h)	Pupae (*n*=10, μl/h)	*K. oxytoca* (μl/h)	Negative control
0	0	0	0	0	0
1	0	0	0	-	0
2	0	0	0	-	0
4	0	0	0	-	0
8	0	0	0	-	0
16	0	0	0	-	0
48	-	-	-	1.84	0

### Nitrogen from urea is largely reabsorbed by host tissues aided by gut symbiotic bacteria

In nature, vertebrate excreta, including bird droppings and mammalian urine, is considered the primary resources providing carbohydrates and nitrogen for tephritid adults. Uric acid and urea are the predominant forms of nitrogen waste in these food sources. In addition, the concentration of urea in maggoty fruits increased as the larvae grow (Additional file 1: Fig. S3) [42, 43]. *B. dorsalis* lack the capacity to convert urea into usable forms of nitrogen (Additional file 1: Table S6), it has been posited for *B. dorsalis* that extracellular gut symbionts recycle such nitrogen waste, converting recycled nitrogen into essential amino acids that are acquired by hosts.

**Fig. 3:**
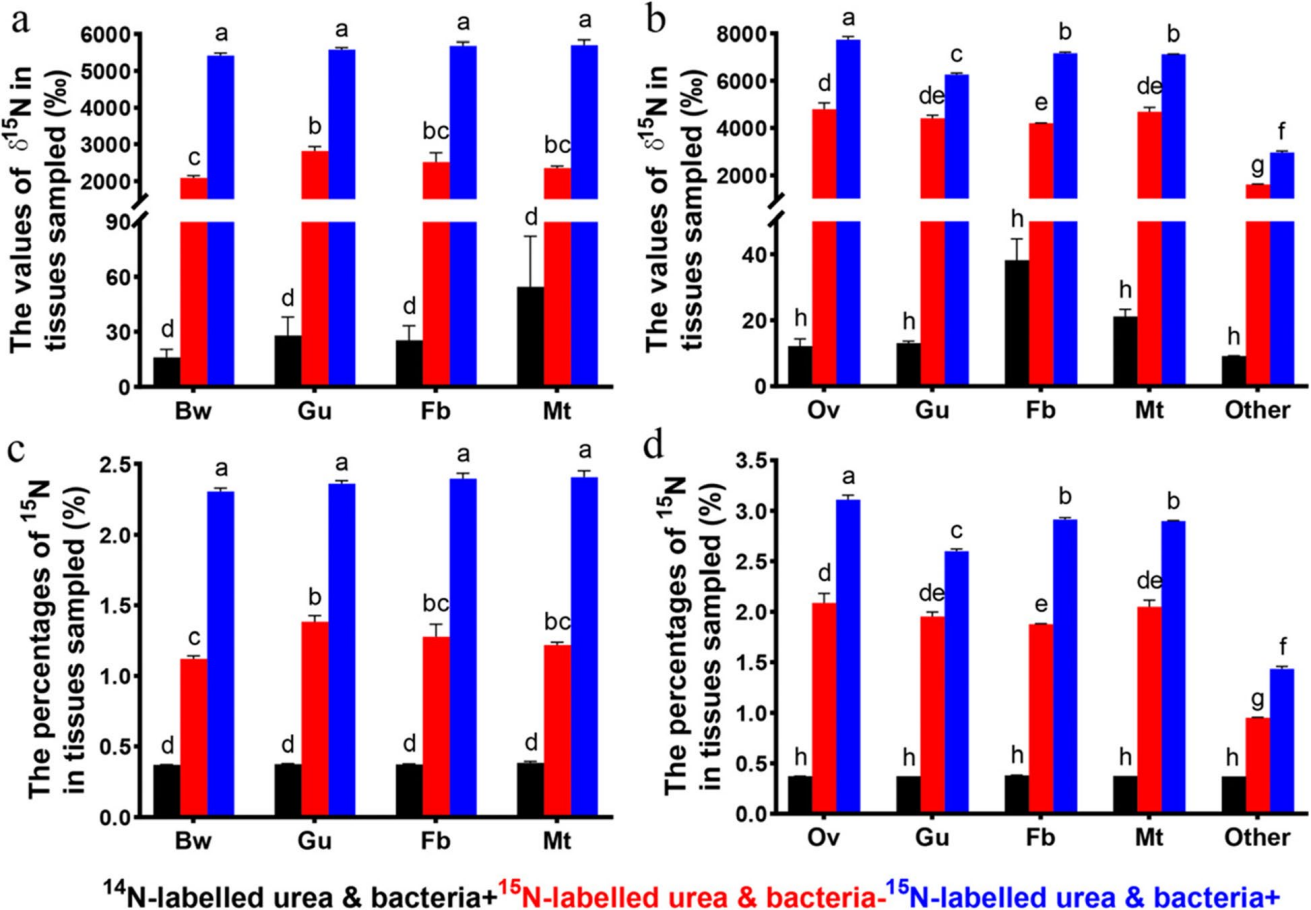
Feeding trial. Bacterial suppression reduces the values of δ^15^N and the percentages of ^15^N in different tissues of *B. dorsalis* consuming ^15^N-labelled urea. **a** The values of δ^15^N in larvae; **b** the values of δ^15^N in adults; **c** the percentages of ^15^N in larvae; **d** the percentages of ^15^N in adults. Bw: body wall; Gu: gut; Fb: fat body; Mt: Malpighian tubules; Ov: ovary; Other: a mixture of head, thorax, wing and leg tissues of adult flies; ^14^N-labelled urea & bacteria+: Flies feeding on ^14^N-labelled urea; ^15^N-labelled urea & bacteria+: Flies feeding on ^15^N-labelled urea; ^15^N-labelled urea & bacteria–: Flies feeding on ^15^N-labelled urea and antibiotics. Differences among means were determined by ANOVA followed by Tukey’s HSD test. Groups not annotated with the same letter show a significant difference (*p* < 0.05)

To test this hypothesis, ^15^N isotope-labelled urea was added to artificial diets to study the process of urea nitrogen assimilation mediated by bacteria. Isotope ratio mass spectrometry measurements of selected tissues of adults and larvae suggested that the values of δ^15^N and the percentages of ^15^N in different tissues associated with insects consuming a diet containing light (^14^N) urea were significantly lower than those of insects feeding on diets containing heavy (^15^N) urea, whether antibiotic treated or untreated, regardless of tissue type or life stage (*p* < 0.001; Fig. 3a–d). The antibiotics used in our study are suitable for suppressing most bacteria (Additional file 1: Fig. S4) [44]. The comparison of the ^15^N-labelled urea & bacteria+ and ^15^N-labelled urea & bacteria– groups suggested the significant incorporation of ^15^N into different tissues of both adults and larvae (*p* < 0.001; Fig. 3a–d). This result indicated that bacterial suppression by antibiotics reduces the level of urea assimilation, so symbiotic bacteria in *B. dorsalis* should be an important driving force for urea recycling.

### Strains isolated from the gut of the host may play important roles in urea metabolism

**Fig. 4:**
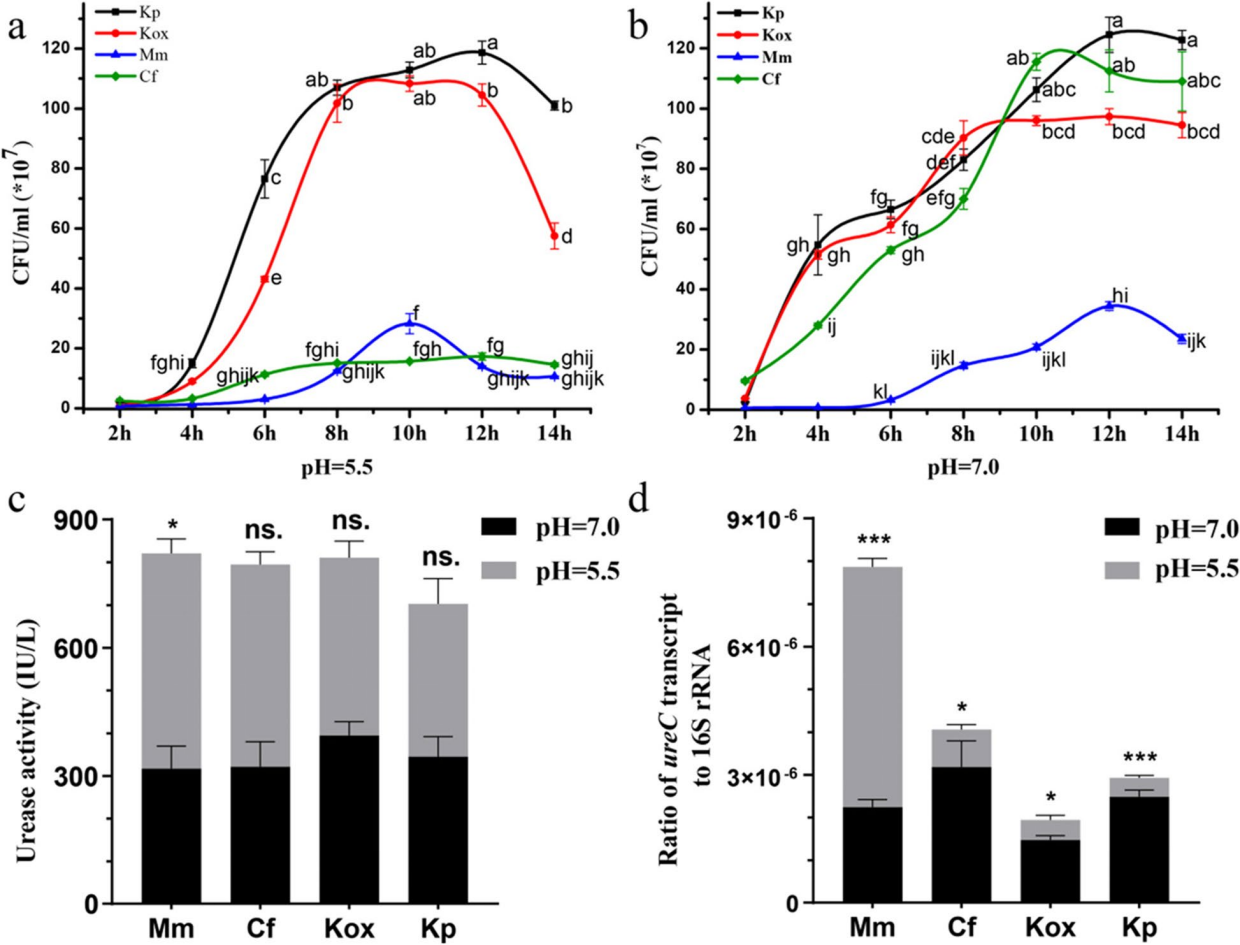
Urease assays. **a**,**b** Bacteria-growth curves were determined at different pH values (**a** pH=5.5, **b** pH=7.0). **c** Urease activity determination of specific bacteria under different pH values (IU/L: international active units per 1 L bacterial suspension). **d** The ratio of bacterial *ureC* to 16S rRNA transcripts. Kp: *K. pneumoniae*; Kox: *K. oxytoca*; Cf: *C. freundii*; Mm: *M. morganii*. Error bars represent the standard error of the mean. Different letters indicate statistical significance (*p* < 0.05). “*,” “**,” and “***” represent statistical significance at *p* < 0.05, 0.01 and 0.001, respectively

To further identify the key functional bacteria mediating the process of urea nitrogen assimilation, *M. morganii*, *K. oxytoca*, and *K. pneumoniae* were isolated from the larval gut and *Citrobacter freundii* was isolated from the adult gut using a Christensen agar base, in which urea was the sole nitrogen source. Given that the ecological niche of the larvae, in which the pH tends to be low, is very different from adult living conditions, urease activity assays of these bacteria were carried out in vitro under acidic (pH=5.5) and neutral conditions (pH=7.0). The bacterial growth curve indicated that, compared with growth under acidic conditions, the stationary phase of these strains was delayed under neutral conditions, except for *K. oxytoca*. *C. freundii* is particularly well suited for growing in medium at pH=7.0. The proliferation ability of *K. pneumoniae*, *K. oxytoca*, and *M. morganii* was comparatively stable at different pH values (Fig. 4a, b). Urease assays showed that the urease activity of *M. morganii* under acidic conditions was significantly higher than that under neutral conditions, whereas there was no significant difference observed in *K. oxytoca*, *K. pneumoniae*, and *C. freundii* (Fig. 4c). The ratio of bacterial *ureC* to 16S rRNA transcripts was determined by real-time quantitative PCR (qPCR), and the results suggested that the ratio of *M. morganii* at low pH was significantly higher than that under neutral conditions. In contrast, the ratios of *K. pneumoniae*, *K. oxytoca*, and *C. freundii* were significantly higher under neutral conditions (Fig. 4d). In summary, the kinetics and enzymatic activities of some isolates vary according to pH values. It seems that *M. morganii* is better suited to function under acidic conditions, whereas *K. oxytoca*, *K. pneumoniae*, and *C. freundii* perform better under neutral conditions.

### Comparison of the completeness of the N_2_ fixation and urea hydrolysis pathways mediated by gut symbiotic bacteria

**Fig. 5:**
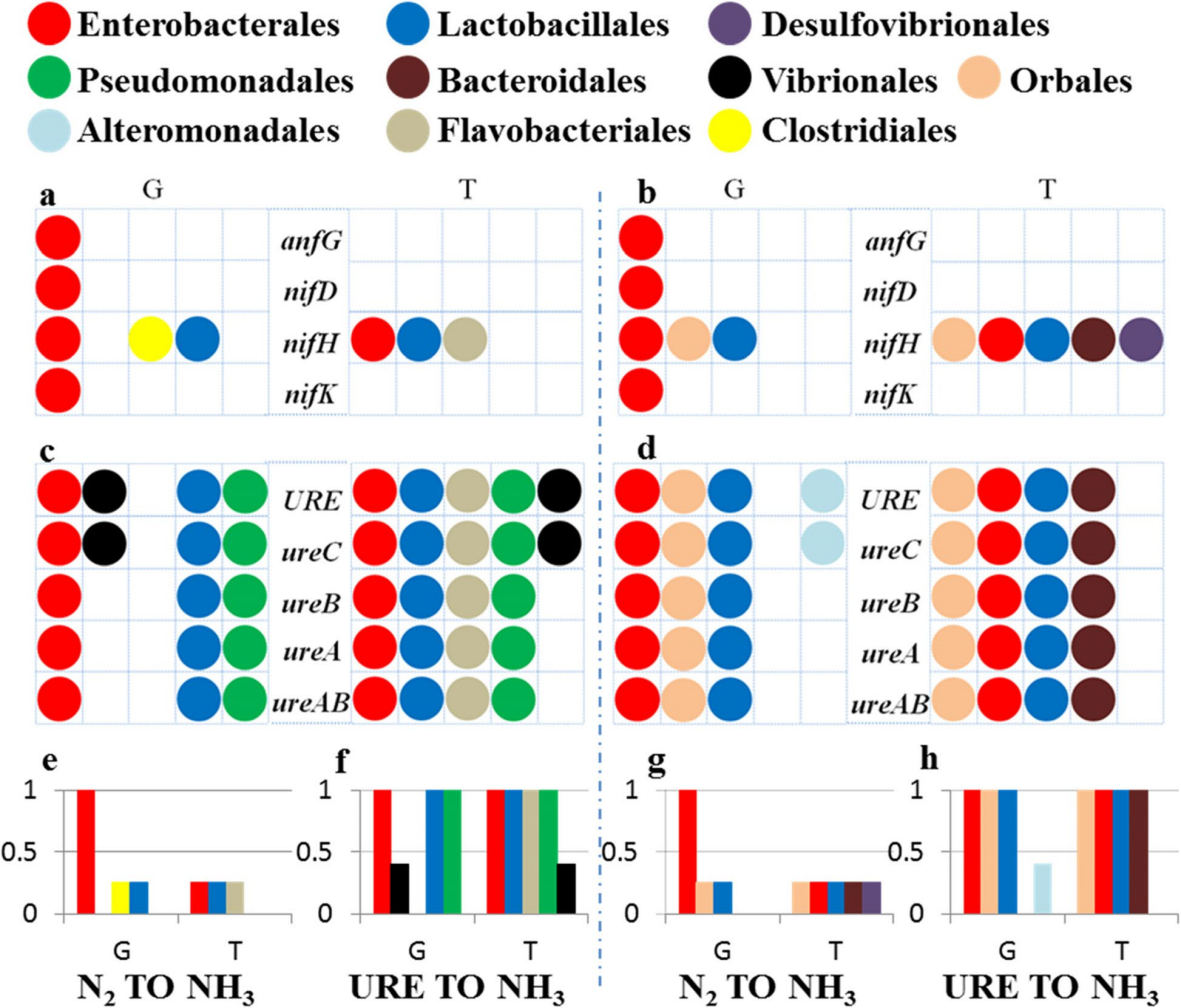
Pathways of nitrogen assimilation mediated by core symbiotic bacteria in *B. dorsalis*. Nitrogen assimilation reactions in larvae are presented on the left of the broken blue line, and those in adults are presented on the right. All the genes involved in each of the reaction steps are listed at the centre of the tables. Core symbiotic bacteria were matched with these genes based on the DNA-seq (G) and RNA-seq (T) annotation results. The bar graphs (**e**–**h**) represent the functional contributions of bacteria in related pathways (i.e., proportions of steps involving bacteria among the total reaction steps). **a** (larvae) and **b** (adults) represent the reactions involved in BNF; **c** (larvae) and **d** (adults) represent the reactions involved in urea hydrolysis

To explain at the genetic level why the nitrogenous waste recycling (NWR) strategy, instead of the BNF strategy, might be the main nitrogen acquisition pathway in *B. dorsalis*, we compared the completeness of the N_2_ fixation and urea hydrolysis pathways mediated by gut symbiotic bacteria in this part. Atmospheric nitrogen can be fixed via the nitrogenase enzyme complex by some bacteria [45, 46]. Three genetically distinct subtypes of nitrogenase (Nif, Vnf, and Anf) with different metal contents are known to play a role in BNF. The most common subtype is the Fe/Mo-type (encoded by *nifD*, *nifH*, and *nifK*), while the Fe/V-type (encoded by *vnfD*, *vnfG*, *vnfH*, and *vnfK*) and Fe/Fe-type (encoded by *anfG*) are infrequent across diazotrophic bacteria [45, 47]. The Functional Ontology Assignments for Metagenomes (FOAM) database was used to map nitrogen fixation genes to symbionts. The functional contributions of core bacteria (Additional file 1: Table S3 and S5, the first five orders of DNA/RNA-seq in larval and adult stages) were presented in the proposed model (Fig. 5). FOAM-based annotation revealed that Fe/V-type coding genes were absent in the nine metagenome libraries. The *anfG*, *nifD*, and *nifK* genes were mapped to the order Enterobacterales in larvae and adults (Fig. 5a, b). In contrast to the limitation of the abovementioned genes to a subset of Enterobacterales, *nifH* in the larval symbiotic bacteria genomes was mapped to the orders Enterobacterales, Clostridiales, and Lactobacillales. Additionally, *nifH* in the adult symbiotic bacterial genomes was mapped to the orders Enterobacterales, Orbales, and Lactobacillales (Fig. 5a, b). However, none of the nitrogenase-encoding genes could be assigned to any bacteria in the nine metatranscriptomics libraries, with the exception of the *nifH* gene (i.e., Enterobacterales, Lactobacillales, and Flavobacteriales in larvae; Orbales, Enterobacterales, Lactobacillales, Bacteroidales, and Desulfovibrionales in adults). The incompleteness of the transcriptional expression of the nitrogen fixation gene cluster indicated a deficiency of dinitrogenase (encoded by *nifD* and *nifK*) in our samples, thus completely disrupting the BNF reaction by blocking electron transfer from dinitrogenase reductase (encoded by the *nifH* gene) to dinitrogenase [48]. These findings indicate that BNF does not occur in any stage of *B. dorsalis*.

Similar to the above analysis method, we evaluated the pathway completeness and functional contributions of bacteria in urea hydrolysation catalysed by urease. Urease is a multisubunit metalloenzyme encoded by a homologous gene cluster (i.e., *URE*, *ureA*, *ureB*, *ureC*, and *ureAB*). According to our sequencing data, all urease-encoding genes existed in each of the metagenomic and metatranscriptomics libraries and were mapped to core symbiotic bacteria found in larvae and adults (Fig. 5c, d). The proportions of the steps involving bacteria among the total reaction steps are presented in Fig. 5e–h. The results indicate that the core bacteria in the gut of *B. dorsalis* possess tremendous potential for urea hydrolysis.

### The synthesis of most essential amino acid (EAA) is potentially mediated by core bacteria

**Fig. 6:**
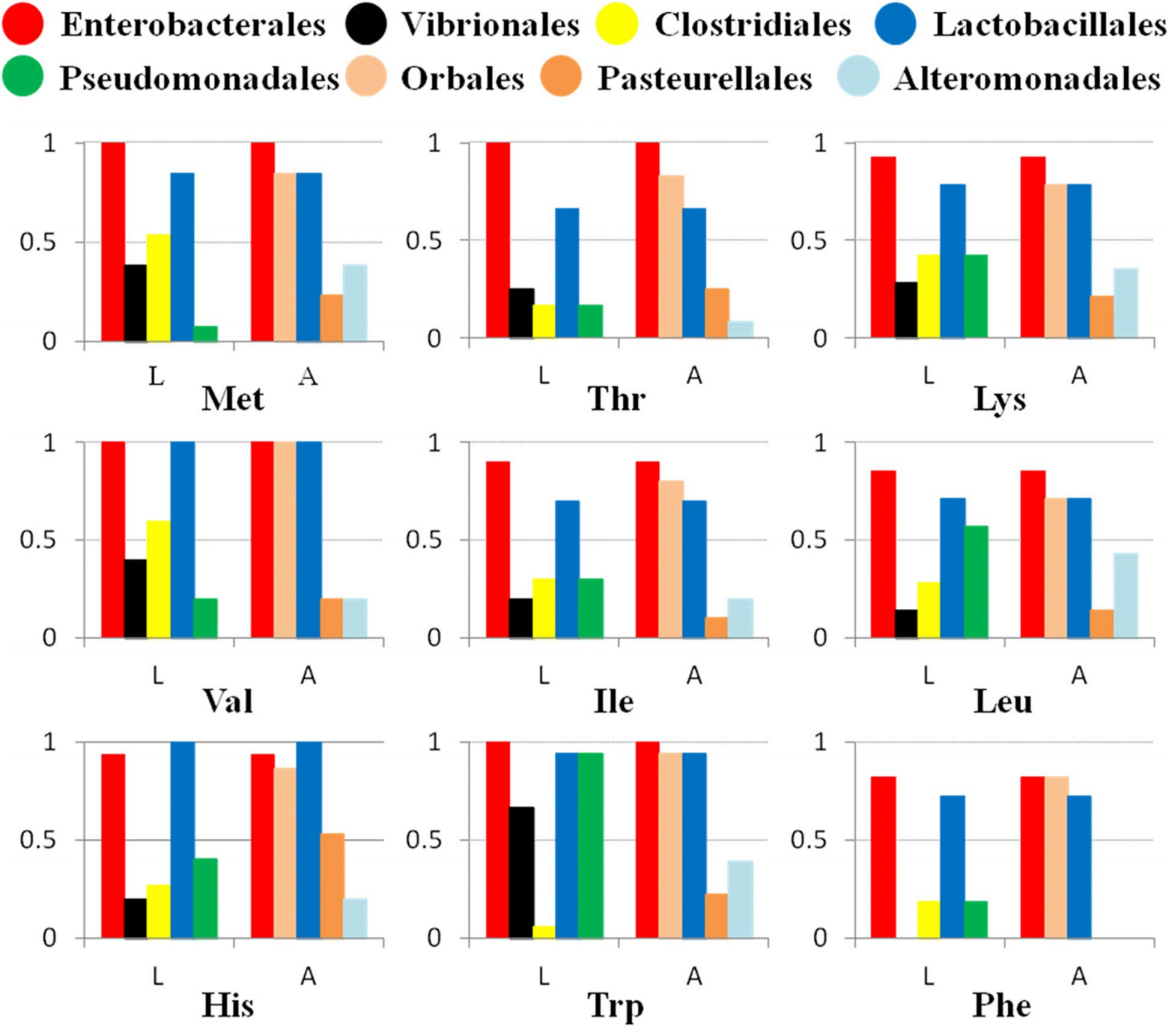
Functional contributions of core bacteria to the biosynthesis of essential amino acids (except for arginine). The bar graphs represent the functional contributions of bacteria in related pathways (i.e., proportion of steps involving bacteria among the total reaction steps) based on the annotation results of the DNA-seq of larvae (L) and adults (A)

How *B. dorsalis* gut symbiotic bacteria assimilate ammonia from urea hydrolysis into their own tissues? Glutamic acid and aspartic acid, which are indispensable ammonium-based donors in the reactions of EAA synthesis, can be derived from ammonium in the presence of glutamate dehydrogenase or oxidoreductases (Additional file 1: Fig. S5). In the current study, the metabolic synthesis pathways of ten EAAs (valine, leucine, isoleucine, histidine, methionine, threonine, lysine, phenylalanine, tryptophan, and arginine), involving 115 specific genes, were constructed (Additional file 1: Fig. S6). The results showed that the core bacterial genomes of adults and larvae retained complete or almost complete pathways for the biosynthesis of all EAAs except arginine (Additional file 1: Fig. S6, Additional file 2). In the arginine synthesis reaction, N-acetylornithine carbamoyltransferase, encoded by *argF*, was not assigned to any symbiont taxa in larvae and adults. EAA synthesis pathways in larvae are mainly mediated by the orders Enterobacterales and Lactobacillales, while those in adults are mainly mediated by the orders Enterobacterales, Orbales, and Lactobacillales (Fig. 6).

## Discussion

Our data demonstrate that nitrogenous waste in maggoty citrus fruits gradually accumulates as larvae develop and can be reabsorbed by insect hosts with the assistance of gut symbiotic bacteria. Bioinformatics analysis also provided cues that gut symbiotic bacteria are hidden players in most EAA biosynthesis pathways by recycling nitrogenous waste. In this process, *M. morganii*, *K. oxytoca*, and other urease-positive bacteria may play important roles in urea metabolism. Our study supports a model in which bacteria utilize nitrogenous waste and produce EAAs as a mechanism of NWR.

Generalist insects usually establish a diverse array of symbiotic interactions with their symbionts to overcome multiple environmental stresses [49]. Our results revealed great differences in the composition and diversity of bacterial communities across different life stages that might be attributed to different ecological niches, in which specialized bacteria are needed to cope with specific pressures of survival. We found that the diversity of the bacterial communities found in adults was higher than that in larvae and pupae, which may be related to the broader scope of foraging and activity patterns in adult flies. At the phylum level, Proteobacteria and Firmicutes showed the highest abundance across all life stages, which was in accord with our previous work based on 16S rRNA amplicon sequencing [50]. However, differences in representative orders and genera are found in different studies [50–52], which might be mainly attributed to the different analysis techniques employed [53]. In addition, factors such as fly age, collection sites, seasons, nutritional status, and host range influence the composition of gut symbionts [37, 51, 54, 55]. Considering different goals, metagenomic methods can recover near-complete genomes of the dominant microbial organisms in communities, and metatrancriptomic data could further reveal important genes and pathways related to their functions. That is why there is a major lack of concordance between the metagenomics and metatranscriptomic datasets. In RNA-seq, the family Orbaceae (mainly represented by *Gilliamella apicola*, *Orbus hercynius*, *Frischella perrara*) was abundant in adult flies. In previous studies, *G. apicola* has been shown to play important roles in pectin and toxic sugar degradation in the honeybee gut [56–58]. Recent studies on the functions of bee gut symbionts in host health demonstrated that short-chain fatty acids were produced by *G. apicola* under low-oxygen conditions and strikingly improved gut physicochemical traits and bee health [59]. *O. hercynius* strains show the highest 16S rRNA gene sequence identity to *G. apicola* (93.9% sequence identity) [60]. *F. perrara* can stimulate the immune system by causing scab formation in the gut of its honeybee host to protect against pathogen infection [61, 62]. *Citrobacter* sp. in the gut of adult flies enhances *B. dorsalis* resistance to the organophosphate insecticide trichlorphon [63]. Therefore, we speculated that *G. apicola* in *B. dorsalis* may be responsible for degrading polysaccharides and providing energy for gut symbionts and insect hosts; *F. perrara* and *C. freundii* may be involved in host resistance. This may explain why KOs for carbohydrate or energy metabolism and drug resistance (antimicrobial) are abundant in adult flies.

The results of the feeding assay showed that nitrogen from urea can be largely absorbed by host tissues aided by symbiotic bacteria. However, the values of δ^15^N and ^15^N% in different tissues of *B. dorsalis* fed ^15^N-labelled urea and antibiotics were significantly increased than those in flies fed ^14^N-labelled urea. Two factors may be responsible for this result. First, although the antibiotics used in our study are efficient in suppressing most symbiotic bacteria (Additional file 1: Fig. S4), completely removing bacteria from the host is impossible, so a fraction of bacteria existing in the guts of the host may constantly contribute to urea recycling. Second, in *Bactrocera* species, the presence or absence of gut symbiotic bacteria affects their foraging behaviour and nutrient ingestion [64, 65]. In particular, our previous studies showed that suppressing the gut bacteria of *B. dorsalis* resulted in a significant increase in food ingestion [66]. For this reason, flies treated with antibiotics consume a greater amount of diets containing ^15^N-labelled urea. Low levels of symbiotic bacteria together with abundant ^15^N-labelled urea intake cause the values of δ^15^N and ^15^N% in flies fed ^15^N-labelled urea and antibiotics to be higher than those of flies fed ^14^N-labelled urea.

The pattern of the NWR is more economical than that of the BNF. It was demonstrated that at least 16 ATP molecules are consumed during the reduction of a single N_2_ molecule [67]. Moreover, the limitations of nitrogenase, including extreme oxygen sensitivity, poor catalytic activity and complicated components also weaken the universal applicability of BNF in most living organisms [46, 67]. By comparison, NWR appears to be a relatively economical investment in many organisms [68]. Taking uric acid degradation in termites as an example, the net energy consumption in the de novo synthesis of one molecule of uric acid is only two molecules of MgATP. Furthermore, 11 additional molecules of MgATP can be produced during uric acid metabolism [69]. Interestingly, acetylene reduction activity associated with the adults of Queensland fruit flies and Mediterranean fruit flies was detected in previous studies [7, 36, 70]. The nitrogen fixation capacity varies according to the emergence days of flies and the mode of treatment (feeding or not feeding diazotrophic bacteria). The author proposed that (1) general experimental conditions such as temperature and relative humidity during the study influenced fly-associated nitrogenase activity, and (2) only when the concentration of ammonia in the gut of relatively well-fed flies dropped to sufficiently low levels would the nitrogenase enzyme complex be switched on [36]. A similar conclusion has been confirmed in termites, in which abundant nitrogen in the diet represses the activity of BNF to reduce energy consumption [25]. In our study, NWR was indicated to be a feasible mechanism of nutrient provisioning for oriental fruit flies, whereas BNF may be inhibited because the transcription of some critical genes is missing. The existence of potential diazotrophic bacteria, such as *K. oxytoca*, is not the determining factor in inducing BNF in *B. dorsalis*. Based on the above analyses, we predict that if nitrogen nutrients can be appropriately supplemented by NWR, the BNF strategy would be suspended temporarily. This pattern may be a general strategy of tephritid fly larvae developing in fruit pulp, but more work is needed to elucidate the scope of the application of NWR.

*Morganella morganii* was the most abundant bacterium found in larvae and is capable of catalysing the urea degradation reaction. In medicine, *M. morganii* is considered an important opportunistic bacterial pathogen that causes a wide range of postoperative wound and urinary tract infections [71]. Metabolites isolated from *M. morganii* exhibit insecticidal effects and are highly toxic to the larvae of Mexican fruit flies, mosquitoes, and wax moths [72–74]. A comparative genomic analysis showed that *M. morganii* contains the urease gene cluster *ureABCEFGD*, and urease is maximally activated in vitro under low pH conditions (pH=5.5) [75, 76]. Our data showed for the first time that *M. morganii* may be involved in recycling nitrogenous waste products and providing metabolizable nitrogen to larvae, together with *K. oxytoca* and other urease-positive strains. This might explain why *M. morganii* was the most abundant bacteria in the *B. dorsalis* larval gut. Furthermore, our results provide clues about how *M. morganii* and nitrogenous waste could be exploited in mass rearing to replace brewer’s yeast and peptone as protein sources. This may achieve significant cost savings in extending sterile insect technique (SIT) procedures in future [77–79].

The gut microbiota is a critical factor driving the development of frugivorous insects within fruit pulp. Genes related to nitrogen assimilation (BNF or NWR) are missing in *B. dorsalis* (Additional file 1: Table S6). In this context, gut symbiotic bacteria become a potent tool in resource competition among frugivorous larvae. Egg surface bacteria are transferred to fruit by the oviposition behaviour of the female parent. Host fruit provides bacteria and newly hatched larvae with suitable metabolizable substrates and developmental conditions (e.g., humidity and pH levels). During larval growth, bacterial communities become established and proliferate within fruit pulp, causing rapid deterioration of the fruit host [41, 80]. In this process, the content of metabolizable nitrogen drops dramatically with nitrogenous waste accumulation. Nitrogen assimilation and EAA synthesis mediated by symbionts will be accelerated when nitrogen nutrition is not sufficient to support larval growth. In short, based on our data, we support a model in which female flies spread bacteria in the host fruit, and the host fruit perpetuates the fly-associated bacterial communities. These bacteria contribute to nitrogen exploitation and enable frugivorous larvae to develop successfully in a confined niche.

## Conclusions

Based on the fact that genes related to BNF or NWR are missing in *B. dorsalis*, (1) we conclude that symbiotic bacteria may contribute to nitrogen transformation in the larvae of *B. dorsalis* in the enclosed environment of fruit pulp; (2) we propose that the pattern of NWR is more likely to be accepted by *B. dorsalis* due to its energy conservation and mild reaction conditions; and (3) we highlight the potential contributions of *M. morganii*, *K. oxytoca*, and other urease-positive strains in hydrolysing nitrogenous waste and providing metabolizable nitrogen for *B. dorsalis*.

## Methods

### Sample collection and handling

Adults, second-instar larvae, and pupae of *B. dorsalis* were collected from the same citrus orchard (China, 25° 36′ 2″ N and 111° 36′ 14″ E) in September 2018 for metagenomics and metatranscriptomics analyses. Adults were trapped with a sweep net, larvae were collected from newly infested fruits, and pupae were produced by allowing matured third-instar larvae collected from fallen fruits to pupate in sterile sand in the laboratory. Prior to gut dissection, adults and larvae were starved for 24 h to eliminate transient microbiota. The whole guts of these specimens were isolated under a light microscope in a laminar flow hood, followed by body surface sterilization in 75% ethanol for 3 min and rinsing three times in sterile distilled water. The intestinal tissues used for DNA or RNA extraction were stored at −80°C for up to 3 months.

### Metagenomics analysis (DNA-seq)

Prior to metagenomic analysis, 35 individual guts isolated from the same developmental stage of *B. dorsalis* were pooled for each replicate. Each life stage (adult, larval, and pupal) included three replicates. DNA was extracted and purified using a Mag-Bind Soil DNA Kit (Omega Bio-Tek, GA, USA) following the manufacturer’s instructions. The quality of the harvested DNA was ascertained by 1.20% agarose gel electrophoresis, and the concentration of double-stranded DNA (dsDNA) was measured using a Quant-iT PicoGreen dsDNA Assay Kit (Thermo Fisher Scientific, MA, USA) in a TBS-380 fluorometer (Turner Biosystems, CA, USA). The purified high-molecular-weight DNA samples were sent to Personalbio Company (Shanghai, China) for whole-genome shotgun (WGS) sequencing. DNA samples were sheared into smaller fragments randomly, and adaptors were ligated to the end-repaired DNA fragments [81]. Nine sequencing libraries were generated across three developmental stages, and high-throughput sequencing was then performed using paired-end 2 × 150 bp reads on an Illumina HiSeq X-ten platform.

Data were first filtered according to quality using FastQC (Website link: http://www.bioinformatics.babraham.ac.uk/projects/fastqc/). Adaptors and low-quality regions (reads containing any ambiguous bases or showing a Phred score below 20) were identified and clipped by using Cutadapt (v1.2.1). On average, 13,327 kb of clean data were generated from each of the metagenomic libraries after removing the trimmed reads with lengths below 50 bp. The reference sequences of *B. dorsalis* downloaded from NCBI GenBank (accession number: NW_011876400.1) were used to eliminate host genetic contamination from the subsequent bioinformatics analysis using Kneaddata v0.7.2 (http://huttenhower.sph.harvard.edu/kneaddata), and removed Host Clean datasets were generated at this step. These removed host clean datasets were assembled de novo by MEGAHIT (https://hku-bal.github.io/megabox/) with the default parameter of k-mer sizes of 27 to 127; therefore, contigs/scaffolds were constructed and assessed based on a De Bruijn graph (Additional file 1: Table S1) [82]. Scaftigs were generated after splitting ambiguous bases within the scaffolds, and the Cluster Database at High Identity with Tolerance (CD-HIT) was then used for further clustering (identity threshold of 99%), and redundancy elimination, thus producing longer and nonredundant gene catalogues [83]. The abundance of scaftigs in each of the metagenomic libraries was calculated after mapping the corresponding clean data to the nonredundant gene catalogues using BWA (http://bio-bwa.sourceforge.net/) [84]. Scaffolds/scaftigs longer than 300 bp were used for gene prediction and open reading frame (ORF) identification by MetaGeneMark (http://exon.gatech.edu/GeneMark/metagenome) [85]. The scaffold sequences of the gut microbiome of *B. dorsalis* larvae, pupae, and adults were deposited in the SRA (Sequence Read Archive) database under accession number PRJNA763789. Nonredundant protein sequence databases were generated after size selection, clustering (identity threshold of 90%) and redundancy elimination by CD-HIT. The abundance of the harvested protein sequences was evaluated according to the results regarding the abundance of corresponding scaftigs in each of the metagenomic libraries using soap.coverage (http://soap.genomics.org.cn/).

All the assembled scaffolds/scaftigs were compared against the reference sequences of bacteria, archaea, fungi, and viruses in the NCBI-NT database (E-value < 0.001). The lowest common ancestor algorithm [86] was applied for sequence annotation and classification using MEGAN [87]. The distributions of species relative abundances in each taxonomic category were calculated based on the abundance information of scaffolds/scaftigs in the samples. Further gene functional annotation was performed with the nonredundant protein sequence databases against public databases, including KEGG, EggNOG, CAZy, Nr, Swiss-Prot, and GO, with an e-value < 0.001.

### Metatranscriptomics analysis (RNA-seq)

The same numbers of samples and replicates indicated above were prepared for RNA-seq. Column Stool RNAout (TIANDZ, Beijing, China) was employed for RNA extraction and purification following the manufacturer’s recommendations. The quality of the harvested RNA was verified using an Agilent 2100 Bioanalyzer (Agilent Technologies, CA, USA) and an agarose gel electrophoresis system. Qualified total RNA samples were subjected to mRNA enrichment and rRNA removal treatment using the Ribo-off rRNA Depletion Kit (Vazyme Biotech, Nanjing, China), and cDNA was then synthesized using mRNA as a template. The WGS sequencing and quality control procedures applied to the raw data were similar to those in the metagenomics analysis. BWA (http://bio-bwa.sourceforge.net/) was used to remove host reads from the clean datasets. Then, residual rRNA sequences were eliminated again with SortMeRNA (http://bioinfo.lifl.fr/RNA/sortmerna/) [88]. Clean data were assembled de novo with Trinity (http://trinityrnaseq.github.io/) to generate full-length transcript sequences [89, 90]. The scaffold sequences of the gut microbiome of *B. dorsalis* larvae, pupae, and adults were deposited in the SRA (Sequence Read Archive) database under accession number PRJNA763809. Unigene sets were constructed after all the transcripts were merged, and redundancy was removed using CD-HIT with 95% identity and 90% minimum coverage. Sequences of mRNA were mapped to unigene sets using Bowtie2 (v2.2.9) with the default parameters [91], and the expression levels of all the unigenes in each of the samples were calculated with RSEM (v1.3.0) [92]. Further transcript functional annotation and downstream analysis were synchronized with the metagenomics analysis.

After the taxonomic classification of transcripts and gene annotation, the FOAM database was used to map functional genes to the microbiota. Simultaneously, the nitrogen metabolic pathways of *B. dorsalis* were manually constructed according to the KEGG pathway database. Ultimately, the functional contributions of bacteria from the insect gut were analysed.

### Acetylene reduction assay (ARA)

The ARA reaction converts acetylene to ethylene, which is catalysed by nitrogenase. Enzyme activity is quantified based on ethylene production, which is detected by gas chromatography [7]. Before the experiment, we prepared closed glass vessels (30 mL) containing wet cotton and adult flies (*n* = 6), second-instar larvae (*n* = 10), pupae (*n* = 10), or the *Klebsiella oxytoca* strain inoculated on nitrogen-free agar slants (in triplicate samples). Empty vessels were used as controls. Acetylene was injected into these vessels at a final concentration of 20% (v/v) by the replacement of an identical volume of air. One hundred microlitres of the air mixture samples from each vessel was injected into a gas chromatography-flame ionization detector (GC-FID, 7890B series, Agilent Technologies) to identify the contents of acetylene and ethylene after 0, 1, 2, 4, 8, and 16 h of incubation at 30 °C [17].

### Feeding trial

Urea, the end-product of purine and uric acid metabolism in vivo and the precursor substance in ammonium assimilation, was chosen for use in the feeding trial. Newly emerged flies were fed sugar and sterile distilled water for 3 days prior to the experiment to eliminate weak flies. After 3 days, the flies were separated by sex to avoid nitrogen loss through oviposition behaviour, and they were then split into three treatment groups of 60 flies each. Three biological replicates were set in each treatment group. The artificial diets were prepared as described previously [33] with slight modifications (Additional file 1: Table S7), where sucrose and urea were employed as the sole carbon and nitrogen sources, respectively. In the first treatment, flies were provided with 20% sucrose water with a mineral mixture, in addition to 0.2274% (weight/volume) unlabelled urea (i.e., mostly ^14^N, recorded as ^14^N-labelled urea & bacteria+). The second treatment group used 5.17 atom% ^15^N-labelled urea (Shanghai Chemical Industry Institute, Shanghai, China) in place of unlabelled urea (recorded as ^15^N-labelled urea & bacteria+). The last group was subjected to antibiotic feeding by providing flies with sugar meal containing ^15^N-labelled urea and antibiotic solutions (3 μg/mL norfloxacin and 5 μg/mL ceftazedime, recorded as ^15^N-labelled urea & bacteria−) [66]. One millilitre of diet was provided in a 6-cm petri dish containing sterilized filter paper (the container was changed every day). Flies were fed three times a day for 1 month. After that, different tissues of adults, including the gut, ovary, fat body, Malpighian tubules, and the rest of the body (i.e., head, thorax, wings and legs) were dissected and stored at −80 °C for further analysis.

The same experimental design was applied to the larvae. Six millilitres of a semisolid defined diet (Additional file 1: Table S7) was provided in a 6-cm petri dish, in which thirty first-instar larvae (newly hatched, 3 days old) were inoculated. After 9 days of feeding, the larvae were dissected to separate the tissues of interest (body wall, gut, fat body, and Malpighian tubules). After all samples were fully dried (65 °C for 48 h in an oven), the values of δ^15^N and the percentages of ^15^N (%) in different tissues sampled were determined with a combined Isoprime 100 stable isotope ratio mass spectrometer with an Elementar Vario EL Cube analyser (Elementar Trading Co., Ltd., Germany) [93]. The results were compared using one-way ANOVA with the dietary treatment as a factor, and the values of δ^15^N and the percentages of ^15^N as dependent variables, followed by Tukey’s HSD test. All data were analysed using SPSS 16.0 (SPSS Inc., IL, USA).

Calculation formulas for δ^15^N and ^15^N (%) [11, 28]:

$$ {\displaystyle \begin{array}{l}{\updelta}^{15}\mathrm{N}\ \left(\permille \right)=1000\ \left[\left\{\left({}^{15}{\mathrm{N}}_{\mathrm{sample}}{/}^{14}{\mathrm{N}}_{\mathrm{sample}}\right)/\left({}^{15}{\mathrm{N}}_{\mathrm{standard}}{/}^{14}{\mathrm{N}}_{\mathrm{standard}}\right)\right\}-1\right]\\ {}{}^{15}{\mathrm{N}}_{\mathrm{standard}}{/}^{14}{\mathrm{N}}_{\mathrm{standard}}=0.003676\\ {}{}^{15}{\mathrm{N}}_{\mathrm{sample}}\left(\%\right)=100\ \left[{}^{15}{\mathrm{N}}_{\mathrm{sample}}/\left({}^{15}{\mathrm{N}}_{\mathrm{sample}}{+}^{14}{\mathrm{N}}_{\mathrm{sample}}\right)\right]\end{array}} $$

### Quantitative urease assay of specific bacteria under different pH values

The urea hydrolysation process catalysed by urease-positive strains converts urea to ammonia and carbonate. Four urease-positive strains of Enterobacterales were isolated from the midguts of *B. dorsalis* larvae and adults using a Christensen agar base. Here, bacterial urease activity was investigated. First, a single colony of the above bacteria was inoculated into 5 mL of Christensen medium. After shaking at constant temperature (37 °C, 180 r/min) for 2, 4, 6, 8, 10, 12, or 14 h, the colony-forming units (CFU) in 1 mL of the resulting solution were recorded by the dilution plate counting method. Moreover, four strains cultured in Christensen medium (pH=5.5/7.0, 10 h) were centrifuged, washed, and diluted with 0.01 M phosphate-buffered saline to harvest bacterial suspensions at equal concentrations (OD600=0.5). One millilitre of the bacterial suspension was sampled for urease activity determination with an enzyme-linked immunosorbent assay (ELISA) kit (Meimian Biotechnology Co. Ltd., Jiangsu, China) following the manufacturer’s instructions after cell disruption by an ultrasonic processor. Three biological replicates were performed for each strain. Finally, we compared the ratio of the bacterial transcripts of *ureC*, which encodes a urease structural protein, to those of 16S rRNA in the 12th hour of growth in Christensen medium. The primer pairs used for qPCR analysis were as follows [94–96]: *ureC* (F) 5′- TGGGCCTTAAAATHCAYGARGAYTGGG- 3′ and *ureC* (R) 5′- GGTGGTGGCACACCATNANCATRTC- 3′; 1114F 5′- CGG CAACGAGCGCAACCC-3′ and 1275R 5′- CCATTGTAGCACGTGTGTAGCC-3′. The relative gene expression data were analysed using the Formula 2^- {Ct (*ureC*)- Ct (16S rRNA)}^. Enzymatic activity assay and the relative *ureC* gene expression level between acidic and neutral conditions were evaluated with *t*-tests at *α* = 0.05.

## Supplementary Information

**Additional file 1: Supplemental methods, figures and tables referenced in the text**. **Figure S1**. Annotation results of secondary metabolic pathways based on KEGG; **Figure S2**. Nitrogen content and mean N:C ratio of different samples; **Figure S3**. Qualitative and quantitative analyses of urea and uric acid by enzymatic colorimetry; **Figure S4**. Dilution coating plate method and qPCR for estimating antibiotic efficacy; **Figure S5**. Nitrogenous waste degradation and EAA biosynthesis pathways constructed based on the KEGG database; **Figure S6**. Pathways for EAAs biosynthesis; **Table S1**. Statistical table of metagenomic assembly (DNA-seq); **Table S2**. Alpha diversity metrics calculated at the 97 % identity level (DNA-seq-based analysis); **Table S3**. Distribution of dominant species (%) in different taxonomic categories based on DNA-seq; **Table S4**. Statistical table of metatranscriptomics assembly (RNA-seq); **Table S5**. Distribution of dominant species (%) in different taxonomic categories based on RNA-seq; **Table S6**. The distribution of some functional genes in *B. dorsalis*; **Table S7**. Nutrient composition of the defined diets used in feeding trial.

**Additional file 2. **Genes from N-metabolic pathways in *B. dorsalis* gut microbiota and their distribution in different microbes.

## Data Availability

All data generated or analysed during this study are included in this published article, its supplementary information files and publicly available repositories. All the raw sequencing reads were deposited in the Sequence Read Archive under BioProjects PRJNA763789 [97] and PRJNA763809 [98].

## Abbreviations

BNF: Biological nitrogen fixation
NWR: Nitrogenous waste recycling
DNA-seq: Metagenomics analysis
RNA-seq: Metatranscriptomics analysis
KOs: KEGG orthologous groups
EAAs: Essential amino acids
ARA: Acetylene reduction assays
SIT: Sterile insect technique
