# Supplementary material for: Gut symbiotic bacteria are involved in nitrogen recycling in the tephritid fruit fly Bactrocera dorsalis
Source: BMC Biol. 2022 Sep 14;20:201. doi: 10.1186/s12915-022-01399-9 (PMC9476588; doi:10.1186/s12915-022-01399-9)
Supplement: Supplementary file 1 — Additional file 1: Supplemental methods, figures and tables referenced in the text. Figure S1. Annotation results of secondary metabolic pathways based on KEGG; Figure S2. Nitrogen content and mean N:C ratio of different samples; Figure S3. Qualitative and quantitative analyses of urea and uric acid by enzymatic colorimetry; Figure S4. Dilution coating plate method and qPCR for estimating antibiotic efficacy; Figure S5. Nitrogenous waste degradation and EAA biosynthesis pathways constructed based on the KEGG database; Figure S6. Pathways for EAAs biosynthesis; Table S1. Statistical table of metagenomic assembly (DNA-seq); Table S2. Alpha diversity metrics calculated at the 97 % identity level (DNA-seq-based analysis); Table S3. Distribution of dominant species (%) in different taxonomic categories based on DNA-seq; Table S4. Statistical table of metatranscriptomics assembly (RNA-seq); Table S5. Distribution of dominant species (%) in different taxonomic categories based on RNA-seq; Table S6. The distribution of some functional genes in B. dorsalis; Table S7. Nutrient composition of the defined diets used in feeding trial. [file 12915_2022_1399_MOESM1_ESM.docx]

**Gut symbiotic bacteria are involved in nitrogen recycling in the tephritid fruit fly *Bactrocera dorsalis***

Xueming Ren^1^; Shuai Cao^1^; Mazarin Akami^1^; Abdelaziz Mansour^1^; Yishi Yang^1^; Nan Jiang^1^; Haoran Wang^1^; Guijian Zhang^1^; Xuewei Qi^1^; Penghui Xu^1^; Tong Guo^1^; Changying Niu^1^*

**Supplementary Experiments**

**Methods**

**Supplementary experiment 1: Detection of uric acid (UA) and urea concentrations in maggoty fruits**

The citrus fruits used in this test were picked from a single tree at Huazhong Agricultural University (30°4′N and 114°3′E) in October 2019. Adults of *B. dorsalis* were trapped in the same citrus orchard and reared in the laboratory for generation expansion. The citrus fruits of the test group were inoculated with 35 newly hatched larvae (2 days) and placed in a constant-temperature incubator (27°C) for 3, 6, and 9 days. Thereafter, citrus pulp (after larvae had been removed) was mashed with a juice extractor, and 10 mL of juice was collected for further analyses. Healthy citrus fruits were set as the control groups, and each group contained three replicates.

Qualitative and quantitative analyses of UA and urea in maggoty fruits were performed using a Uric Acid (UA) Test Kit and Urea Assay Kit purchased from Nanjing Jiancheng Bioengineering Institute (Nanjing, China) following the manufacturer’s instructions. The principle of both kits is coupled enzyme reactions. After catalysis by bioenzymes, UA/urea is converted into substances with distinct colours, which provides important evidence for UA/urea qualitative analysis. The absorbance of these substances is proportional to the concentration of UA/urea at a particular wavelength. As such, the quantitative analysis of UA/urea in samples could be performed based on the standard curves of UA/urea concentration-absorbance. The results were compared using one-way ANOVA followed by Tukey’s HSD test. All data were analysed using SPSS 16.0.

**Supplementary experiment 2: Dilution coating plate method and real-time quantitative PCR (qPCR) for estimating antibiotic efficacy**

In the feeding trial, after one month (adults)/nine days (larvae) of feeding, the guts of adult flies/larvae were dissected to estimate antibiotic efficacy by the dilution coating plate method and qPCR. First, two guts were crushed in 1.5 mL tube, and after 1,000-fold dilution in sterile 0.01 M phosphate-buffered saline, 100 μl of the diluent was homogeneously coated onto a LA plate, which was then incubated in constant temperature incubator (37°C) for 12 h. Second, four guts were dissected and pooled for qPCR analysis, DNA was extracted and purified using a Mag-Bind Soil DNA Kit (Omega Bio-Tek, GA, USA) following the manufacturer’s instructions. Three biological repetitions and four technical repetitions were performed in each treatment. The primer pairs used in qPCR were as follows: 16S rDNA gene primers (F 5’- ACTCCTACGGGAGGCAGCAG- 3’ and R 5’- AT TACCGCGGCTGCTGG- 3’) and β-actin (F 5’- TCGATCATGAAGTGCGATGT- 3’ and R 5’- ATCAGCAATAC CGGGGTACA- 3’). qPCR was performed in a volume of 20 μl. Each mixture consisted of 10 μl of SYBR Green Mix (Bio-Rad), each primer at 100 nM and 5 ng of DNA. The amplification program consisted of (1) preincubation at 95°C for 30 s; (2) 40 cycles of 95°C for 5 s, 55°C for 30 s, and 72°C for 31 s; and (3) one cycle of 95°C for 15 s, 60°C for 1 min and 95°C for 15 s.

**Results**

**Urea is the main component of nitrogenous waste in maggoty citrus fruits**

The concentration of urea in maggoty fruits increased as the larvae grew; in particular, the concentration of urea in the third-instar larval stage (9 d, 9.93±0.67 mmol/L) was significantly higher than that in the first-instar (3 d, *p* < 0.001, 2.29±0.31 mmol/L) or second-instar (6 d, *p* < 0.01, 4.49±0.75 mmol/L) larval stage. However, The concentration of UA in our samples could not be detected (Fig. S3), suggesting that the main component of the nonprotein nitrogen metabolites in maggoty fruits is urea.

**Antibiotics used in our study are suitable for suppressing most bacteria**

In sample diluents inoculated in LA medium, the density of strain colonies treated with antibiotics was lower than that in the groups treated with ^14^N-labelled urea & bacteria+ or ^15^N-labelled urea & bacteria+, which was consistent with the qPCR analysis results (Fig. S4). Therefore, 3 µg/mL norfloxacin and 5 µg/mL ceftazedime work well in suppressing most bacteria in *B. dorsalis*.

**Supplementary Figures**
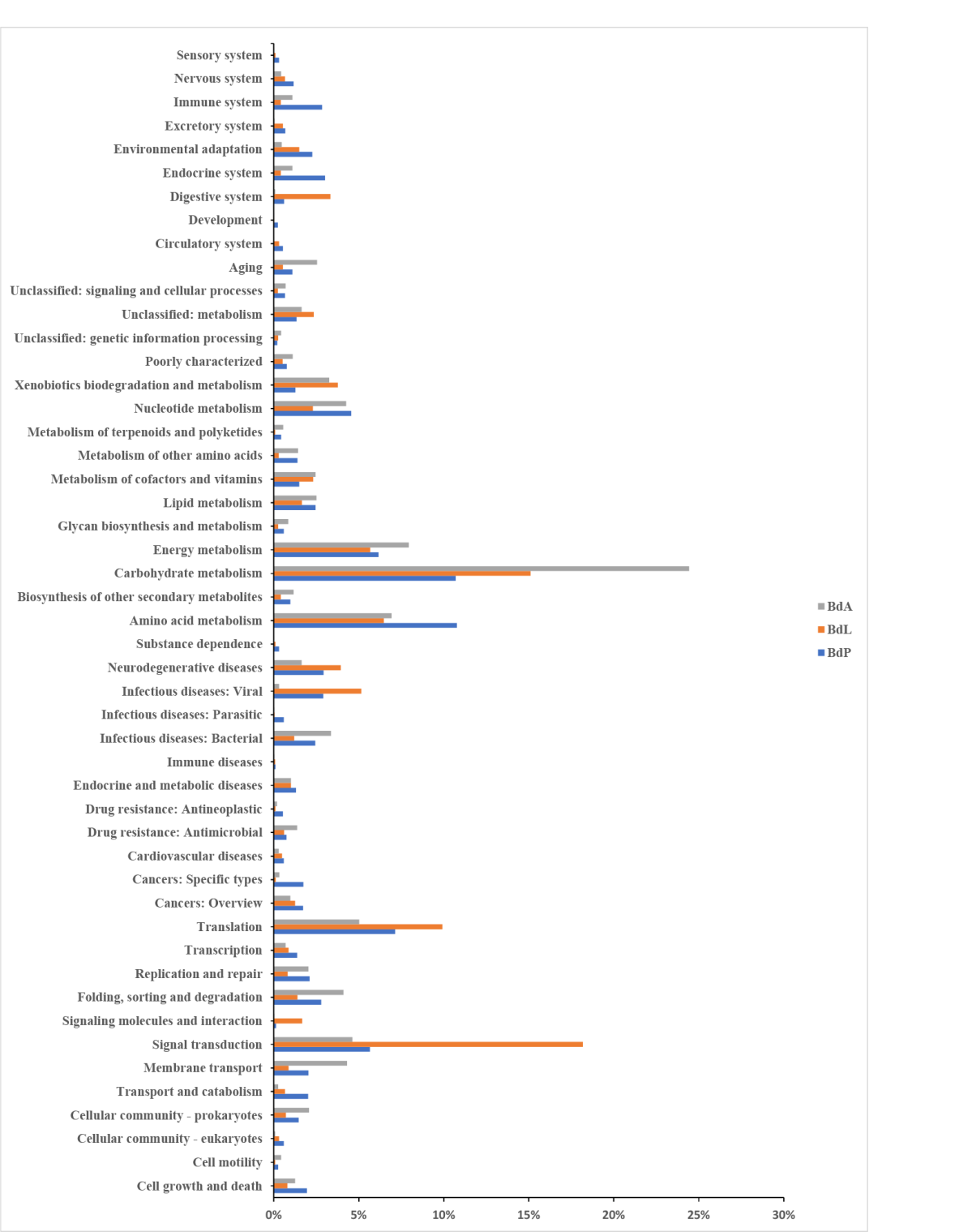


Fig. S1 Annotation results of secondary metabolic pathways based on KEGG. BdA: adults; BdL: larvae; BdP: pupae.


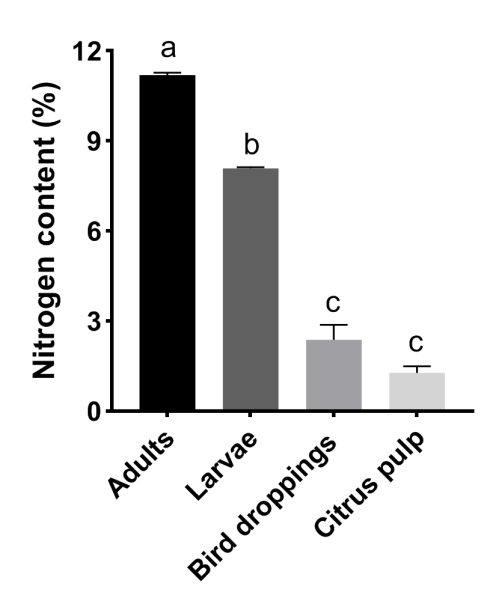

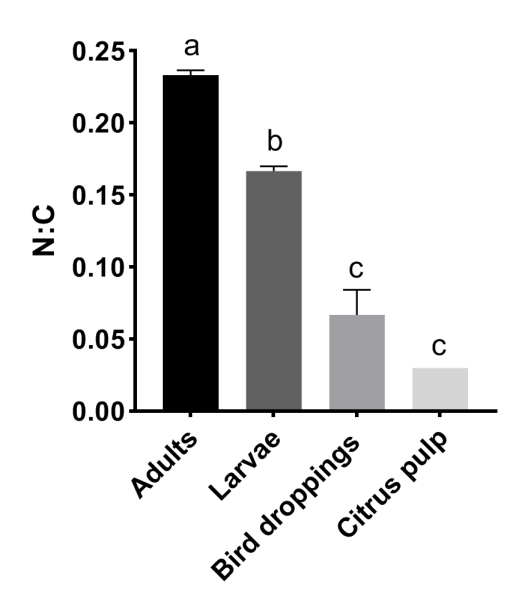


a

b

Fig. S2 Nitrogen content (a) and mean N:C ratio (b) of different samples. Differences among means were determined by ANOVA followed by Tukey’s HSD test. Groups not marked by the same letter show significant differences (*p* < 0.05).


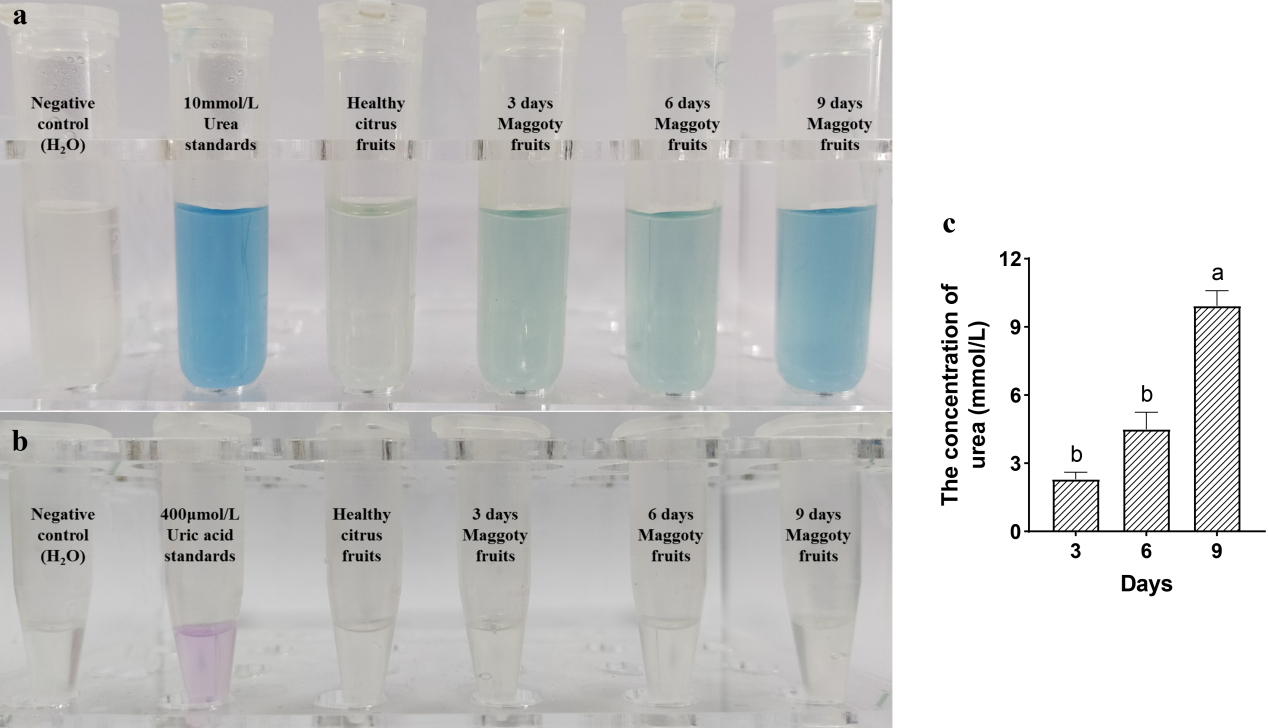


Fig. S3 Qualitative and quantitative analyses of urea and uric acid by enzymatic colorimetry. **a.** [Chromogenic](javascript:;) [reaction](javascript:;) for urea detection; **b.** [chromogenic](javascript:;) [reaction](javascript:;) for UA detection; **c.** concentrations of urea in different samples. Groups not marked by the same letter show significant differences (*p* < 0.05).


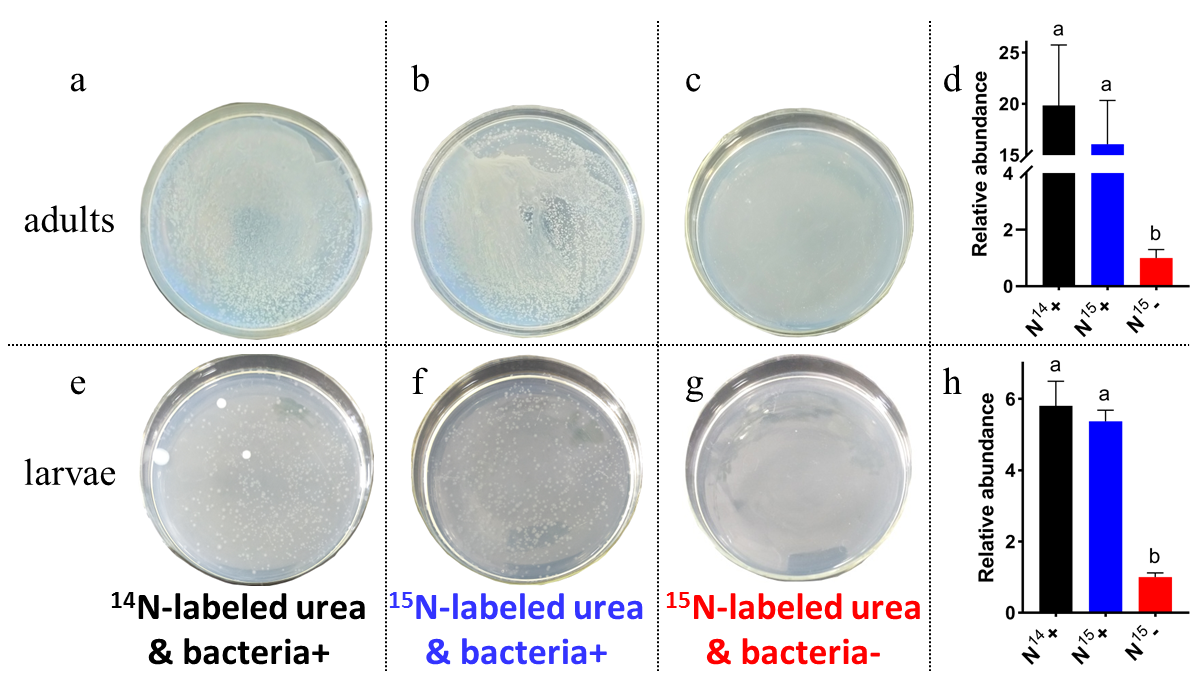


Fig. S4 Dilution coating plate method and qPCR for estimating antibiotic efficacy. **a-d:** Antibiotic efficacy detection in adults; **e-h:** antibiotic efficacy detection in larvae. Groups not marked by the same letter show significant differences (*p* < 0.05).


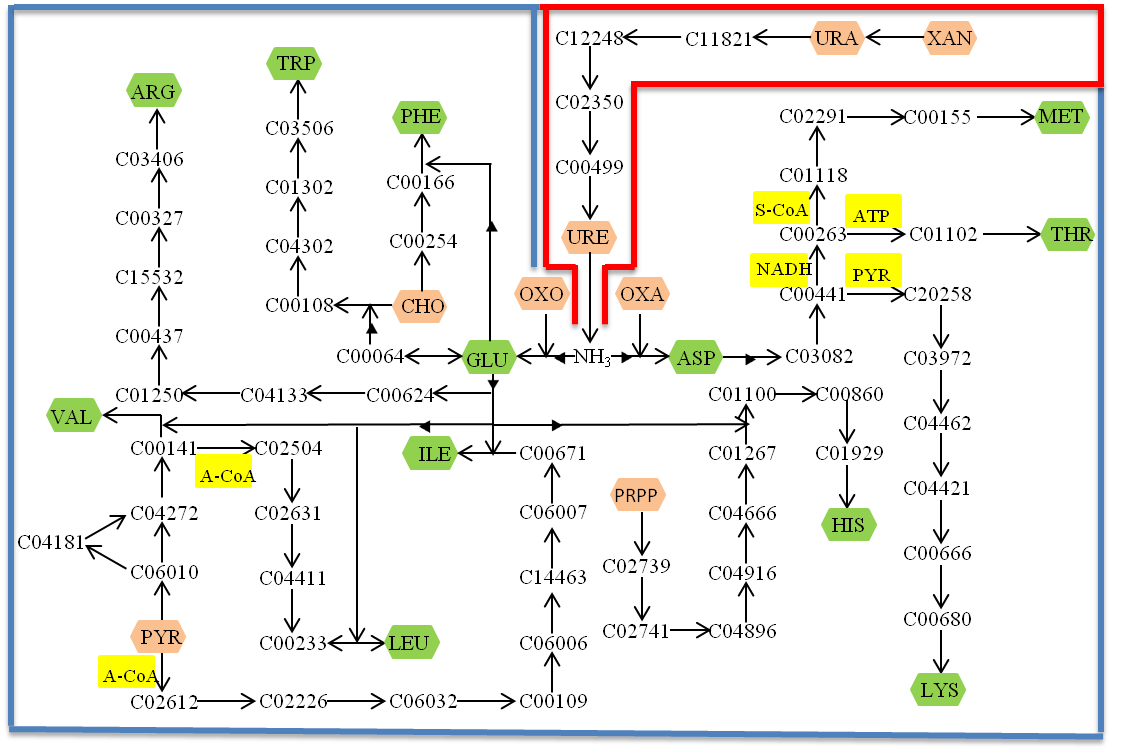


Fig. S5 Nitrogenous waste degradation (red border) and EAA biosynthesis (blue border) pathways constructed based on the KEGG database. Reacting substances and reaction products are indicated with abbreviations or KEGG identifiers. Green shading indicates important amino acids, pink shading indicates important reactants, and yellow shading indicates important enzymes. Solid arrows (i.e., in the middle of a line) indicate the provision of an ammonium group in that step. XAN: xanthine; URA: urate; URE: urea; OXO: 2-oxoglutarate; OXA: oxaloacetate; CHO: chorismate; PYR: pyruvate; PRPP: 5-phospho-alpha-D-ribose 1-diphosphate.


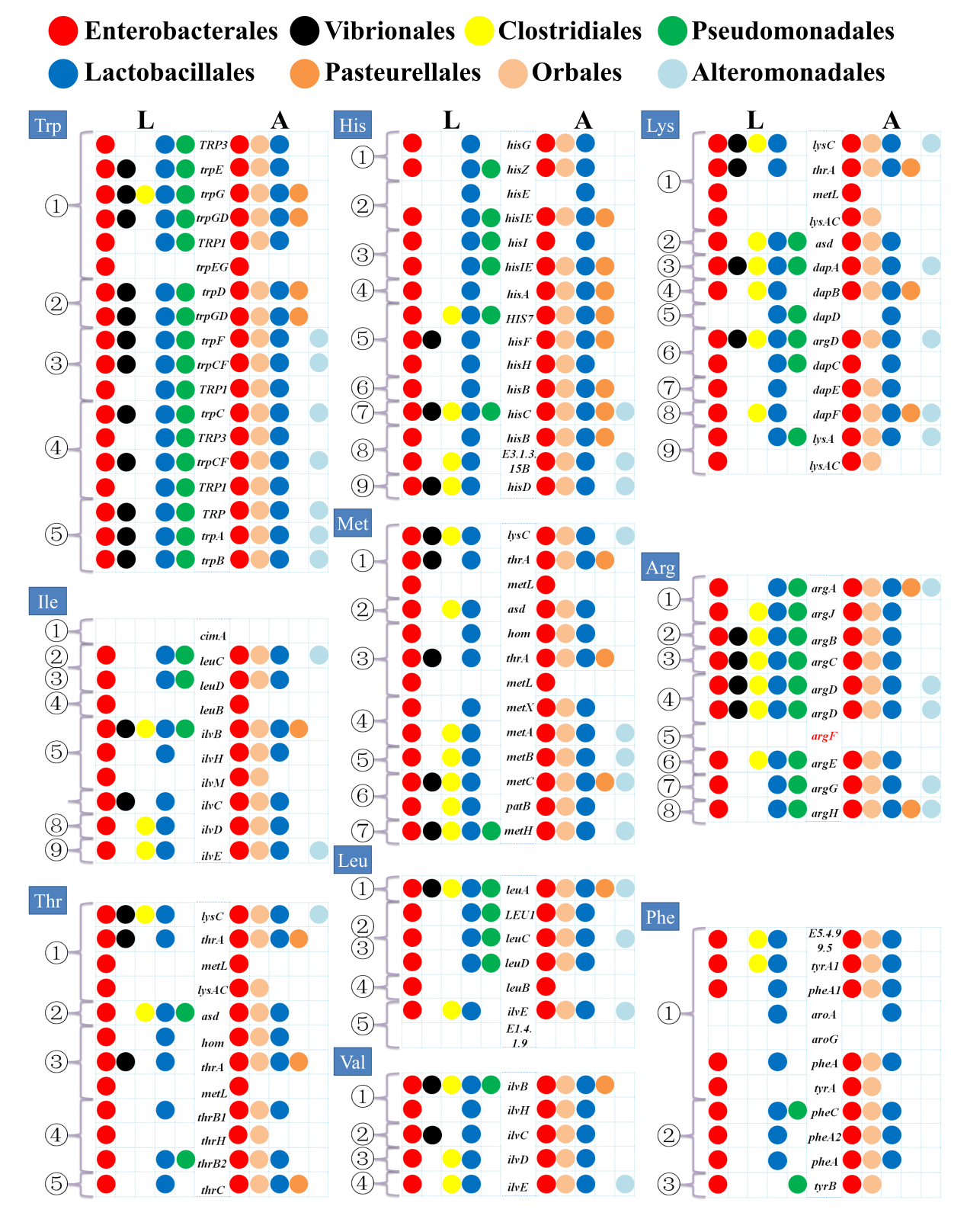
Fig. S6 Pathways for EAAs biosynthesis. EAAs are derived from glutamic acid or aspartic acid by transamination mediated by gut bacteria in *B. dorsalis*. All the genes involved in each of the reaction steps are listed at the centre of the tables. Core bacteria were matched with these genes based on the annotation results of genomic sequences. Genes highlighted in red were not matched with bacteria. L: larvae; A: adults.

**Supplementary Tables**

**Table S1 Statistical table of metagenomic assembly (DNA-seq)**

| Sample name | BdA1 | | BdA2 | | BdA3 | |
| --- | --- | --- | --- | --- | --- | --- |
|  | Contigs | Scaffolds | Contigs | Scaffolds | Contigs | Scaffolds |
| Shortest sequence length | 200 | 200 | 200 | 200 | 200 | 200 |
| Longest sequence length | 86157 | 120206 | 316140 | 316140 | 174029 | 186620 |
| Total numbers of sequences | 319275 | 300136 | 295320 | 277064 | 332112 | 313002 |
| The N50 length | 973 | 1114 | 914 | 1046 | 838 | 933 |
| The N90 length | 349 | 349 | 346 | 345 | 340 | 340 |
| Total numbers of ambiguous bases | 0 | 89353 | 0 | 91057 | 0 | 100312 |
| Total sequence size | 248421405 | 246045420 | 222024910 | 219828369 | 238582238 | 236250069 |
| Total numbers of GC | 113733784 | 112653376 | 99188814 | 98203478 | 105164901 | 104133295 |
| %(G+C) content of the sequences | 45.783 | 45.786 | 44.675 | 44.673 | 44.079 | 44.078 |
| Total numbers of sequences > 1 kb | 51946 | 49123 | 45493 | 42965 | 45599 | 43675 |
| Sample name | BdL1 | | BdL2 | | BdL3 | |
|  | Contigs | Scaffolds | Contigs | Scaffolds | Contigs | Scaffolds |
| Shortest sequence length | 200 | 200 | 200 | 200 | 200 | 200 |
| Longest sequence length | 169146 | 187744 | 60850 | 93060 | 15111 | 18951 |
| Total numbers of sequences | 92853 | 91063 | 118594 | 113453 | 88846 | 87422 |
| The N50 length | 543 | 555 | 762 | 804 | 510 | 517 |
| The N90 length | 329 | 329 | 342 | 342 | 324 | 324 |
| Total numbers of ambiguous bases | 0 | 5132 | 0 | 25067 | 0 | 4083 |
| Total sequence size | 50620906 | 50379115 | 84983260 | 84434980 | 44344682 | 44139530 |
| Total numbers of GC | 18595425 | 18503438 | 37467073 | 37207426 | 15543895 | 15468790 |
| %(G+C) content of the sequences | 36.735 | 36.728 | 44.088 | 44.066 | 35.052 | 35045 |
| Total numbers of sequences > 1 kb | 3917 | 4245 | 12165 | 11457 | 3662 | 4136 |
| Sample name | BdP1 | | BdP2 | | BdP3 | |
|  | Contigs | Scaffolds | Contigs | Scaffolds | Contigs | Scaffolds |
| Shortest sequence length | 200 | 200 | 200 | 200 | 200 | 200 |
| Longest sequence length | 18020 | 24108 | 11304 | 25017 | 7189 | 7511 |
| Total numbers of sequences | 122899 | 119560 | 111618 | 107688 | 109190 | 107162 |
| The N50 length | 550 | 565 | 580 | 597 | 528 | 538 |
| The N90 length | 329 | 329 | 332 | 332 | 328 | 328 |
| Total numbers of ambiguous bases | 0 | 11801 | 0 | 13664 | 0 | 4670 |
| Total sequence size | 65629196 | 65226676 | 62552366 | 62132495 | 55892333 | 55593622 |
| Total numbers of GC | 24729346 | 24569636 | 22959877 | 22794933 | 19868751 | 19759938 |
| %(G+C) content of the sequences | 37.680 | 37.668 | 36.705 | 36.688 | 35.548 | 35.544 |
| Total numbers of sequences > 1 kb | 7043 | 7367 | 7923 | 7889 | 5052 | 5588 |

**Table S2 Alpha diversity metrics calculated at the 97 % identity level (DNA-seq-based analysis)**

| Sample name | Simpson Chao1 | Chao1 | ACE | Shannon |
| --- | --- | --- | --- | --- |
| BdA1 | 0.952719332841 | 2942.83333333 | 2938.63269052 | 5.90695797255 |
| BdA2 | 0.963366507839 | 3001.39130435 | 2832.82589197 | 6.33597551478 |
| BdA3 | 0.965944379266 | 2885.4516129 | 2894.05967359 | 6.46786626882 |
| BdL1 | 0.736969633733 | 593.533333333 | 647.016400809 | 3.18615176862 |
| BdL2 | 0.775431950153 | 1019.27272727 | 778.937745415 | 3.20263346856 |
| BdL3 | 0.862050088634 | 513.6 | 509.582782266 | 3.80779279682 |
| BdP1 | 0.895065686469 | 742.405405405 | 803.484897113 | 4.23621670224 |
| BdP2 | 0.898786785488 | 600.24 | 595.384119107 | 4.19224348506 |
| BdP3 | 0.877735596707 | 583.551724138 | 617.665818752 | 4.14861461526 |

**Table S3 Distribution of dominant species (%) in different taxonomic categories based on DNA-seq**

| Samples | Phylum (%) | Order (%) | Genus (%) |
| --- | --- | --- | --- |
| Adults | Proteobacteria (88.40);  Firmicutes (5.60);  Bacteroidetes (2.58) | Enterobacterales (71.98);  Orbales (6.27);  Lactobacillales (3.79);  Pasteurellales (2.50);  Alteromonadales (1.37);  Flavobacteriales (1.31) | *Citrobacter* (20.93); *Enterobacter* (11.87);  *Klebsiella* (11.26);  *Kluyvera* (3.24);  *Escherichia* (2.82);  *Frischella* (2.78); *Gilliamella* (2.31) |
| Larvae | Proteobacteria (77.53);  Firmicutes (1.08);  Bacteroidetes (0.02) | Enterobacterales (75.33);  Vibrionales (1.17);  Clostridiales (0.92);  Lactobacillales (0.14);  Pseudomonadales (0.07);  Burkholderiales (0.02) | *Morganella* (31.05); *Klebsiella* (20.81);  *Enterobacter* (14.48);  *Vibrio* (1.16); *Clostridium* (0.91); *Citrobacter* (0.87);  *Providencia* (0.48) |
| Pupae | Proteobacteria (65.90);  Firmicutes (1.23);  Bacteroidetes (0.05) | Enterobacterales (61.34);  Vibrionales (3.22);  Clostridiales (0.71);  Lactobacillales (0.46);  Burkholderiales (0.14);  Rhodospirillales (0.07) | *Morganella* (15.40);  *Klebsiella* (15.15);  *Enterobacter* (14.96);  *Providencia* (6.16); *Vibrio* (3.21); *Citrobacter* (1.27);  *Serratia* (0.84) |

**Table S4 Statistical table of metatranscriptomics assembly (RNA-seq)**

| Sample name | BdA1 | BdA2 | | BdA3 | | BdL1 | BdL2 | BdL3 | BdP1 | BdP2 | BdP3 |
| --- | --- | --- | --- | --- | --- | --- | --- | --- | --- | --- | --- |
| Shortest sequence length | 201 | 201 | 201 | | 201 | | 201 | 201 | 201 | 201 | 201 |
| Longest sequence length | 6757 | 10604 | 8038 | | 6993 | | 5115 | 3372 | 4273 | 6301 | 6322 |
| Total numbers of sequences | 88683 | 118389 | 109462 | | 39709 | | 44587 | 30805 | 52626 | 60205 | 57950 |
| The N50 length | 387 | 395 | 391 | | 323 | | 309 | 295 | 337 | 350 | 338 |
| The N90 length | 231 | 231 | 231 | | 223 | | 220 | 218 | 225 | 226 | 224 |
| Total numbers of ambiguous bases | 0 | 0 | 0 | | 0 | | 0 | 0 | 0 | 0 | 0 |
| Total sequence size | 33560543 | 45306681 | 41680651 | | 13211385 | | 14382676 | 9626329 | 17872313 | 21139750 | 19584374 |
| Total numbers of GC | 12359662 | 17049163 | 15563992 | | 4291549 | | 5135214 | 3058229 | 6357378 | 7471585 | 6860166 |
| %(G+C) content of the sequences | 36.828 | 37.631 | 37.341 | | 32.484 | | 35.704 | 31.769 | 35.571 | 35.344 | 35.029 |
| Total numbers of sequences > 1 kb | 2287 | 3109 | 2870 | | 413 | | 388 | 218 | 483 | 887 | 452 |

**Table S5 Distribution of dominant species (%) in different taxonomic categories based on RNA-seq**

| Samples | Phylum (%) | Order (%) | Genus (%) |
| --- | --- | --- | --- |
| Adults | Proteobacteria (58.46);  Firmicutes (16.52);  Bacteroidetes (8.05) | Orbales (23.39);  Enterobacterales (19.12); Lactobacillales (14.33); Bacteroidales (4.59); Desulfovibrionales (3.61);  Flavobacteriales (2.81) | *Gilliamella* (10.40); *Orbus* (5.32); *Lactococcus* (3.47); *Vagococcus* (3.29); *Frischella* (3.06); *Enterococcus* (2.94); *Dysgonomonas* (2.86) |
| Larvae | Proteobacteria (14.27);  Firmicutes (1.24);  Bacteroidetes (0.97) | Enterobacterales (9.74);  Lactobacillales (0.86);  Flavobacteriales (0.68);  Pseudomonadales (0.58);  Vibrionales (0.54);  Rhodobacterales (0.37) | *Morganella* (2.67); *Klebsiella* (1.11); *Enterobacter* (0.62); *Vibrio* (0.43); *Acinetobacter* (0.39); *Chlamydia* (0.32); *Escherichia* (0.30) |
| Pupae | Proteobacteria (10.87);  Firmicutes (0.87);  Bacteroidetes (0.59) | Enterobacterales (6.29);  Lactobacillales (0.69);  Vibrionales (0.49);  Rhodobacterales (0.42);  Flavobacteriales (0.41);  Pseudomonadales (0.23) | *Providencia* (2.82);  *Morganella* (1.09);  *Vibrio* (0.40);  *Roseobacter* (0.27);  *Enterobacter* (0.20);  *Lactococcus* (0.18);  *Enterococcus* (0.17) |

**Table S6** **The distribution of some functional genes in *B. dorsalis*.** It suggests that these genes are missing in the genome of *B. dorsalis*.

| Function | Gene name | Homologous sequence in NCBI | gene length (bp) | Object sequence in local database | Score | E value |
| --- | --- | --- | --- | --- | --- | --- |
| nitrogen fixation | anfG | ID: 3625969 | 351 | Bdor_1477 | 38 | 0.57 |
|  | nifD | ID: 3718049 | 1,482 | Bdor_3421 | 38 | 2.5 |
|  | nifH | ID: 31829083 | 894 | Bdor_1697 | 40 | 0.38 |
|  | nifK | ID: 2686860 | 1,470 | Bdor_881 | 38 | 2.5 |
| Urea degration | URE | ID: 843076 | 4,836 | Bdor_3063 | 44 | 0.13 |
|  | ureC | ID: 32288872 | 1,734 | Bdor_1366 | 38 | 2.9 |
|  | ureB | ID: 899104 | 1,710 | Bdor_2369 | 40 | 0.74 |
|  | ureA | ID: 913824 | 303 | Bdor_4039 | 38 | 0.49 |
|  | ureAB | ID: 1100965 | 684 | Bdor_953 | 38 | 1.1 |

**Table S7 Nutrient composition of the defined diets used in** **feeding trial.**

| Constituents | Amount (g/100ml) | | |
| --- | --- | --- | --- |
|  | ^14^N-labelled urea & bacteria + | ^15^N-labelled urea & bacteria + | ^15^N-labelled urea & bacteria – |
| **Main ingredient** |  |  |  |
| Sucrose | 20.000 | 20.000 | 20.000 |
| Urea(^14^N/^15^N) | 2.274*10^-1^ (N^14^) | 2.274*10^-1^(N^15^) | 2.274*10^-1^ (N^15^) |
| **Minerals and salts** |  |  |  |
| FeSO_4_ | 5.000*10^-3^ | 5.000*10^-3^ | 5.000*10^-3^ |
| MnSO_4_ | 1.260*10^-3^ | 1.260*10^-3^ | 1.260*10^-3^ |
| ZnCl_2_ | 1.260*10^-3^ | 1.260*10^-3^ | 1.260*10^-3^ |
| CuSO_4_ | 6.200*10^-4^ | 6.200*10^-4^ | 6.200*10^-4^ |
| MgSO_4_ | 4.000*10^-2^ | 4.000*10^-2^ | 4.000*10^-2^ |
| KH_2_PO_4_ | 1.693*10^-1^ | 1.693*10^-1^ | 1.693*10^-1^ |
| Ca(H_2_PO_4_)_2_ | 2.000*10^-2^ | 2.000*10^-2^ | 2.000*10^-2^ |
| KCl | 2.340*10^-1^ | 2.340*10^-1^ | 2.340*10^-1^ |
| NaCl | 9.000*10^-2^ | 9.000*10^-2^ | 9.000*10^-2^ |
| **Antibiotics** |  |  |  |
| Norfloxacin | - | - | 3.000*10^-4^ |
| Ceftazedime | - | - | 5.000*10^-4^ |
| Agar (Adults/larvae) | -/1.340 | -/1.340 | -/1.340 |
| DDW | 100.000 | 100.000 | 100.000 |
